# Disruption of the circadian clock drives *Apc* loss of heterozygosity to accelerate colorectal cancer

**DOI:** 10.1126/sciadv.abo2389

**Published:** 2022-08-10

**Authors:** Sung Kook Chun, Bridget M. Fortin, Rachel C. Fellows, Amber N. Habowski, Amandine Verlande, Wei A. Song, Alisa L. Mahieu, Austin E. Y. T. Lefebvre, Jason N. Sterrenberg, Leandro M. Velez, Michelle A. Digman, Robert A. Edwards, Nicholas R. Pannunzio, Marcus M. Seldin, Marian L. Waterman, Selma Masri

**Affiliations:** ^1^ Department of Biological Chemistry, University of California, Irvine, Irvine, CA 92697, USA.; ^2^ Department of Microbiology and Molecular Genetics, University of California, Irvine, Irvine, CA 92697, USA.; ^3^ Department of Biomedical Engineering, University of California, Irvine, Irvine, CA 92697, USA.; ^4^ Department of Medicine, University of California, Irvine, Irvine, CA 92697, USA.; ^5^ Department of Pathology and Laboratory Medicine, University of California, Irvine, Irvine, CA 92697, USA.

## Abstract

An alarming rise in young onset colorectal cancer (CRC) has been reported; however, the underlying molecular mechanism remains undefined. Suspected risk factors of young onset CRC include environmental aspects, such as lifestyle and dietary factors, which are known to affect the circadian clock. We find that both genetic disruption and environmental disruption of the circadian clock accelerate *Apc-*driven CRC pathogenesis in vivo. Using an intestinal organoid model, we demonstrate that clock disruption promotes transformation by driving *Apc* loss of heterozygosity, which hyperactivates Wnt signaling. This up-regulates *c-Myc*, a known Wnt target, which drives heightened glycolytic metabolism. Using patient-derived organoids, we show that circadian rhythms are lost in human tumors. Last, we identify that variance between core clock and Wnt pathway genes significantly predicts the survival of patients with CRC. Overall, our findings demonstrate a previously unidentified mechanistic link between clock disruption and CRC, which has important implications for young onset cancer prevention.

## Teaser

Disruption of the circadian clock accelerates CRC progression by driving *Apc* loss of heterozygosity.

## INTRODUCTION

The canonical Wnt signaling pathway regulates development, differentiation, and proliferation (*1*) and also plays an important role in the pathogenesis of colorectal cancer (CRC) (*2*–*4*). Adenomatous polyposis coli (APC) is a component of the multiprotein destruction complex that is responsible for regulation of β-catenin stability in a proteasome-dependent manner (*5*–*8*). β-Catenin coactivates T cell factor/lymphoid enhancer factor (TCF/LEF)–mediated transcription to regulate Wnt-dependent gene expression (*9*–*12*). APC mutations compromise the activity of the destruction complex, which, in turn, aberrantly activates the Wnt signaling pathway (*2*–*4*). *APC* point mutations, deletions, and loss of heterozygosity (LOH) events have been reported in 80% of human CRC cases (*13*, *14*), and these mutations drive the initiation of intestinal adenoma development (*15*–*20*). In addition, *Apc* mutations are associated with second hits in key oncogenic pathways, including *Kras, Braf, p53,* and *Smad4*, and these mutations drive progression to adenocarcinoma (*21*–*24*). Thus, while *APC* is a critical gatekeeper involved in CRC initiation, other secondary pathways that control signaling, proliferation, and metabolism collectively contribute to disease progression. In addition, this is evidenced by the alarming increase in young onset CRC cases over the last few decades (*25*–*27*). The underlying cause of this rise in young onset CRC is unknown but is attributed to environmental factors, which impinge on intestinal physiology and are linked to the circadian clock.

The molecular clock consists of the core transcription factors, circadian locomotor output cycles kaput (CLOCK) and aryl hydrocarbon receptor nuclear translocator-like protein 1 (*Arntl* encoding BMAL1), which drive the oscillation of tissue-specific gene expression programs (*28*–*30*). The circadian clock maintains cellular homeostasis and systemic physiology (*31*–*33*). However, altered circadian gene expression signatures are associated with several diseases including multiple cancer types (*34*–*42*). Human data from The Cancer Genome Atlas (TCGA) show a significant correlation between a subset of deregulated core clock genes and oncogenic driver pathways, survival rates, and tumor staging in 32 different cancer types (*43*). In addition, human colorectal tumors display disrupted clock gene expression compared to normal intestinal epithelium (*44*, *45*). In further support of the circadian clock playing a vital role in maintaining intestinal homeostasis, the clock has been reported to gate cell cycle progression and regulate Wnt secretion in the intestinal epithelium (*46*), which is important for maintaining intestinal stem cell function (*47*, *48*). Disruption of the circadian clock increased numbers of intestinal polyps driven by the *Apc^Min/+^* model of multiple intestinal neoplasia (*49*, *50*). While these studies implicate the circadian clock in playing an essential role in intestinal homeostasis, the molecular mechanism of how clock disruption accelerates CRC remains undefined.

To address this question, we developed a new tissue-specific genetically engineered mouse model (GEMM) to define the molecular pathways linking circadian disruption and pathogenesis of CRC. Our findings demonstrate that genetic deletion of both intestinal *Apc* and *Bmal1* results in a statistically significant increase in polyp formation versus disruption of *Apc* alone. Using a light shift paradigm, we also demonstrated that environmental disruption of the circadian clock accelerated tumor burden in our *Apc* mutant GEMM. We isolated intestinal crypts and developed organoid cultures from our GEMM to define the underlying mechanism of clock disruption on disease progression. Unexpectedly, digital polymerase chain reaction (dPCR) and whole-exome sequencing (WES) of organoids revealed that disruption of intestinal *Bmal1* accelerated LOH of *Apc.* This, in turn, aberrantly activated Wnt signaling to drive organoid formation and proliferation. We found that *Apc* LOH hyperactivated *c-Myc*, a known Wnt target gene (*51*–*53*), to drive heightened glycolytic metabolism. In addition, using colon tissue to develop patient-derived organoids (PDOs), real-time bioluminescence monitoring revealed that tumor PDOs lose circadian rhythms when compared to normal PDOs from the same patient. Last, we leveraged transcriptomic data from 512 patients with CRC in TCGA, which identified that discordant patterns of variation between circadian genes and the Wnt signaling pathway resulted in poor overall survival. These data highlight the importance of the circadian clock in the intestine and confirm that pathways identified using our GEMM and mouse organoid systems are strongly conserved in humans. Collectively, our data demonstrate that disruption of the circadian clock drives *Apc* LOH to hyperactivate Wnt signaling and enhance MYC-dependent glycolytic metabolism to accelerate CRC progression.

## RESULTS

### Intestinal *Bmal1* disruption accelerates CRC in vivo

**Fig. 1.**
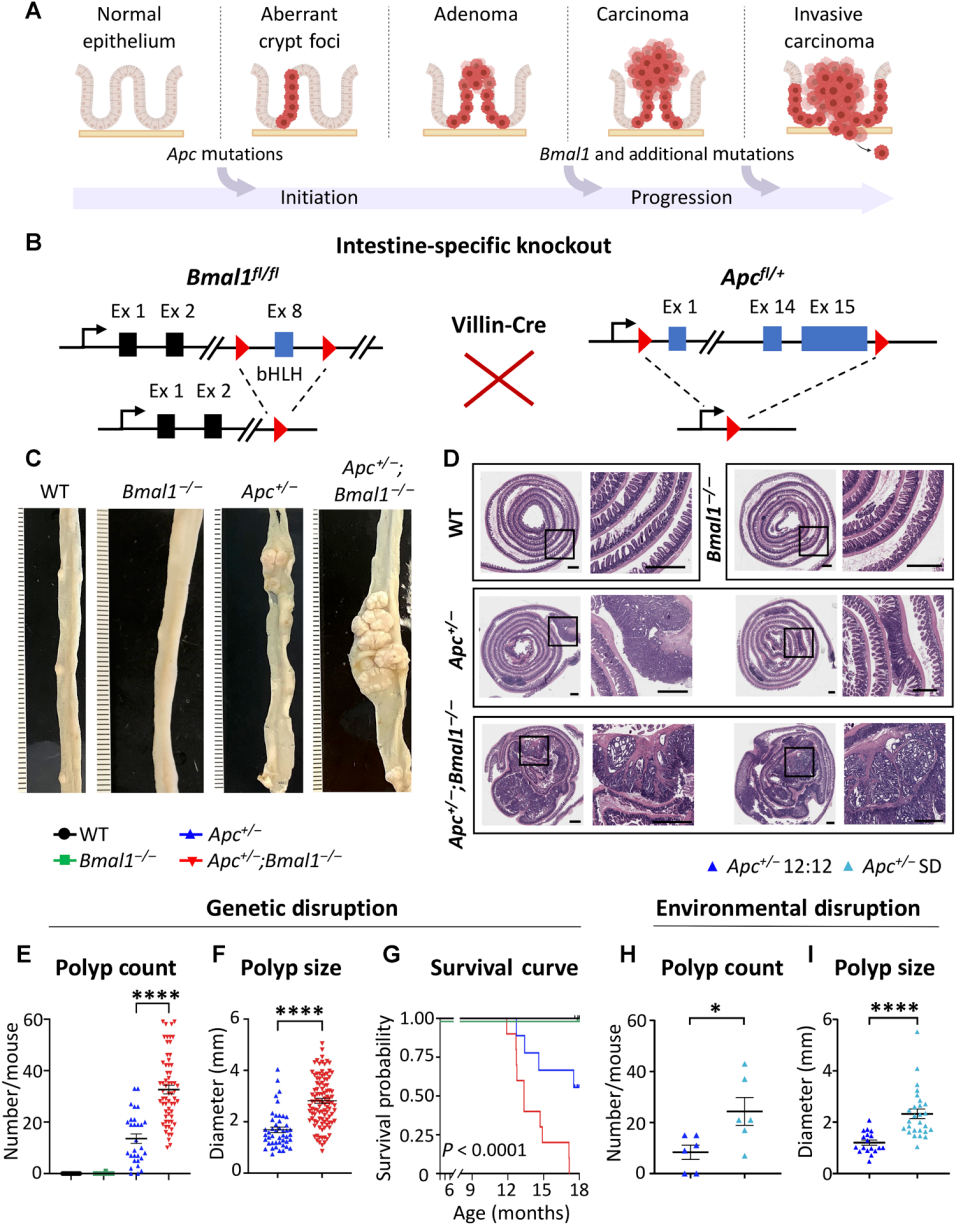
Disruption of circadian clock accelerates intestinal tumorigenesis in vivo. (**A**) Schematic depicting the initiation and progression of CRC through *Apc*, *Bmal1*, and additional mutations. (**B**) In vivo intestine-specific gene targeting strategy for *Bmal1* and *Apc*. (**C**) Linearized ileum tissue from representative mice of all genotypes. (**D**) Overview of representative sections of hematoxylin and eosin (H&E)–stained small intestinal Swiss rolls from WT, *Bmal1*^−/−^, *Apc*^+/−^, and *Apc*^+/−^;*Bmal1*^−/−^ mice. Scale bars, 1 mm. (**E**) Scatterplot of small intestinal polyp count from 30 WT, 30 *Bmal1*^−/−^, 29 *Apc*^+/−^, and 62 *Apc*^+/−^;*Bmal1*^−/−^ mice. (**F**) Scatterplot of individual polyp sizes from the small intestine of *Apc*^+/−^ and *Apc*^+/−^;*Bmal1*^−/−^ mice (*n* = 8 mice per genotype). (**G**) Kaplan-Meier survival curve of 9 to 11 mice per genotype up to 18 months. (**H**) Scatterplot of small intestinal polyp count from six *Apc*^+/−^ mice maintained in 12-hour light:12-hour (12:12) dark conditions and six *Apc*^+/−^ mice maintained in shift disruption (SD) conditions. (**I**) Scatterplot of individual polyp sizes from the small intestine of 12:12 and SD *Apc*^+/−^ mice. Data represent the mean ± SEM, and statistical significance was determined by one-way analysis of variance (ANOVA) with Tukey’s multiple comparison test for (E), log-rank (Mantel-Cox) test for (G), and Student’s unpaired *t* test for (F), (H), and (I). Asterisks represent *P* values from *t* test or multiple comparisons, with *P < 0.05 and ****P < 0.0001.

*Apc* mutations are known to initiate aberrant crypt foci (ACFs) and adenomas (*15*–*20*), and we hypothesized that the additional disruption of the intestinal clock could drive CRC progression through the accumulation of secondary mutations (Fig. 1A). To define the molecular mechanism of how disruption of the circadian clock accelerates CRC, we developed a new tissue-specific GEMM (Fig. 1B). Heterozygotes conditionally floxed for deletion of exons 1 to 15 in *Apc (Apc*^*+/*Δ*ex1–15*^), a model prone to adenoma development (*19*), were crossed with conditionally floxed homozygous *Bmal1^fl/fl^* mice, where the basic helix-loop-helix (bHLH) domain in exon 8 is deleted (*54*). It has been reported that expression of Cre recombinase in *Bmal1^fl/fl^* mice rendered these animals arrhythmic in circadian gene expression in a tissue-specific manner (*54*, *55*), making this an appropriate model to study clock disruption. Using Villin-Cre transgenic mice, *Apc* and *Bmal1* were selectively deleted in intestinal epithelial cells (IECs; *Apc*^*+/*Δ*ex1–15*^*;Bmal1^fl/fl^;Villin-Cre)*. We denote *Bmal1^fl/fl^;Villin-Cre* mice as *Bmal1^−/−^*, *Apc*^*+/*Δ*ex1–15*^*;Villin-Cre* mice as *Apc^+/−^*, and *Apc*^*+/*Δ*ex1–15*^*;Bmal1^fl/fl^;Villin-Cre* mice as *Apc^+/−^;Bmal1^−/−^* or double mutant. Expected genetic deletions, loxP sites, and the presence of Villin-Cre were verified using quantitative PCR (qPCR) of genomic DNA (gDNA) from IECs (fig. S1, A to C).

Unexpectedly, when male and female mice were euthanized at 9 to 10 months of age, greater intestinal polyposis was observed in *Apc^+/−^;Bmal1^−/−^* mice versus *Apc^+/−^* mice (Fig. 1C). Wild-type (WT) and *Bmal1^−/−^* mice did not harbor any polyps, consistent with previous studies reporting that *Bmal1^−/−^* alone is not sufficient to initiate intestinal polyp formation (*54*, *56*). Furthermore, we sectioned and stained Swiss rolls from the small intestine with hematoxylin and eosin (H&E). WT and *Bmal1^−/−^* samples displayed normal small intestinal architecture with uniform crypts, villi, isolated lymphoid follicles, and Peyer’s patches (Fig. 1D). Intestinal H&E sections from *Apc^+/−^* mice contained ACFs, which are early neoplastic precursor lesions, and occasional tubular adenomas (Fig. 1D). Notably, *Apc^+/−^;Bmal1^−/−^* mice showed a marked increase in neoplastic changes, ranging from ACFs and tubular adenomas to locally invasive adenocarcinomas extending into the muscularis propria (Fig. 1D). Microscopic images of intestinal tissue from both *Apc^+/−^* and *Apc^+/−^;Bmal1^−/−^* mice showed typical cytologic signs of neoplasia, with nucleomegaly and prominent nucleoli, hyperchromasia, and increased mitotic figures (Fig. 1D). To further validate our model of *Apc* disruption, we performed immunohistochemistry (IHC) for β-catenin using sectioned intestinal Swiss rolls. Our data demonstrate that β-catenin staining is highly abundant in tumor sections from *Apc^+/−^* and *Apc^+/−^;Bmal1^−/−^* mice versus normal intestinal epithelial staining (fig. S2).

To better define disease burden, a cohort of 151 mice was used for polyp enumeration. A statistically significant increase in intestinal polyp incidence was identified in *Apc^+/−^;Bmal1^−/−^* mice versus *Apc^+/−^* animals (Fig. 1E). Polyp size was also measured in equal numbers of animals per genotype and found to be significantly increased in *Apc^+/−^;Bmal1^−/−^* mice (Fig. 1F). To define sex-specific differences in our GEMM, *Apc^+/−^* and *Apc^+/−^;Bmal1^−/−^* mice were subdivided by sex to assess disease burden. Polyp size was significantly increased in male versus female *Apc^+/−^;Bmal1^−/−^* mice (fig. S1D). Although we observe 100% disease penetrance in the small intestine, colonic polyps were found in 10% of *Apc^+/−^;Bmal1^−/−^* mice, while no polyps were detected in colons of *Apc^+/−^* mice (fig. S1E). As an additional assessment of disease burden and inflammation, we determined spleen weight in control versus tumor-bearing animals. Spleen weight was significantly higher in both *Apc^+/−^* and *Apc^+/−^;Bmal1^−/−^* mice, with the most significant increase in double-mutant animals (fig. S1F). To examine the impact of disease burden on overall survival, age-matched mice from all four genotypes were monitored for life span. Kaplan-Meier survival analysis revealed that *Apc^+/−^;Bmal1^−/−^* mice have significantly reduced survival, and no double-mutant mice were viable beyond 18 months (Fig. 1G).

To determine whether tumor burden affected systemic metabolism, we profiled body weight changes and indirect calorimetry measurements in our GEMM. Body weight decreased with age in *Apc^+/−^;Bmal1^−/−^* mice relative to other genotypes and significantly decreased starting at 10 months of age, when disease burden is pronounced (fig. S1K). Using whole-body composition analysis through magnetic resonance imaging (MRI), we determined both fat mass and lean mass in our GEMM. Fat mass relative to body weight decreased in *Apc^+/−^;Bmal1^−/−^* mice starting at 7 months of age, reaching significance at 9 to 11 months when disease burden is pronounced (fig. S1G). Similarly, there was a significant increase in lean mass relative to body weight in *Apc^+/−^;Bmal1^−/−^* mice at 9 to 11 months of age (fig. S1H). Consistent with sex-specific differences in tumor burden, there was a decreased trend in fat mass and significant increase in lean mass relative to body weight in male mice (fig. S1, I and J). We also performed indirect calorimetry measurements using metabolic cages to determine changes in respiratory exchange ratio (RER) and locomotor activity in our GEMM. We did not observe any major differences in RER and locomotor activity between control and tumor-bearing animals (fig. S3, A and B). Together, our indirect calorimetry analysis indicates that tumor-bearing mice still maintain rhythmic behavior; however, tumor progression significantly affects body weight composition.

To define the impact of circadian environmental disruption on CRC pathogenesis, we subjected WT and *Apc^+/−^* mice to a shift disruption (SD) paradigm, where the light cycle was phase-advanced every other day for 10 weeks. This SD paradigm models night shift work three to four times per week. To validate that the SD paradigm resulted in disrupted circadian rhythms, mice were subjected to indirect calorimetry analysis, and we observed a loss of rhythmic RER, locomotor activity, and food intake (fig. S3, C to I). Our SD paradigm significantly increased both tumor count and size in *Apc^+/−^* mice (Fig. 1, H and I). These data illustrate that *Bmal1* and *Apc* loss acts synergistically to accelerate intestinal polyposis and CRC progression in vivo.

### *Bmal1* disruption accelerates intestinal organoid proliferation ex vivo

**Fig. 2.**
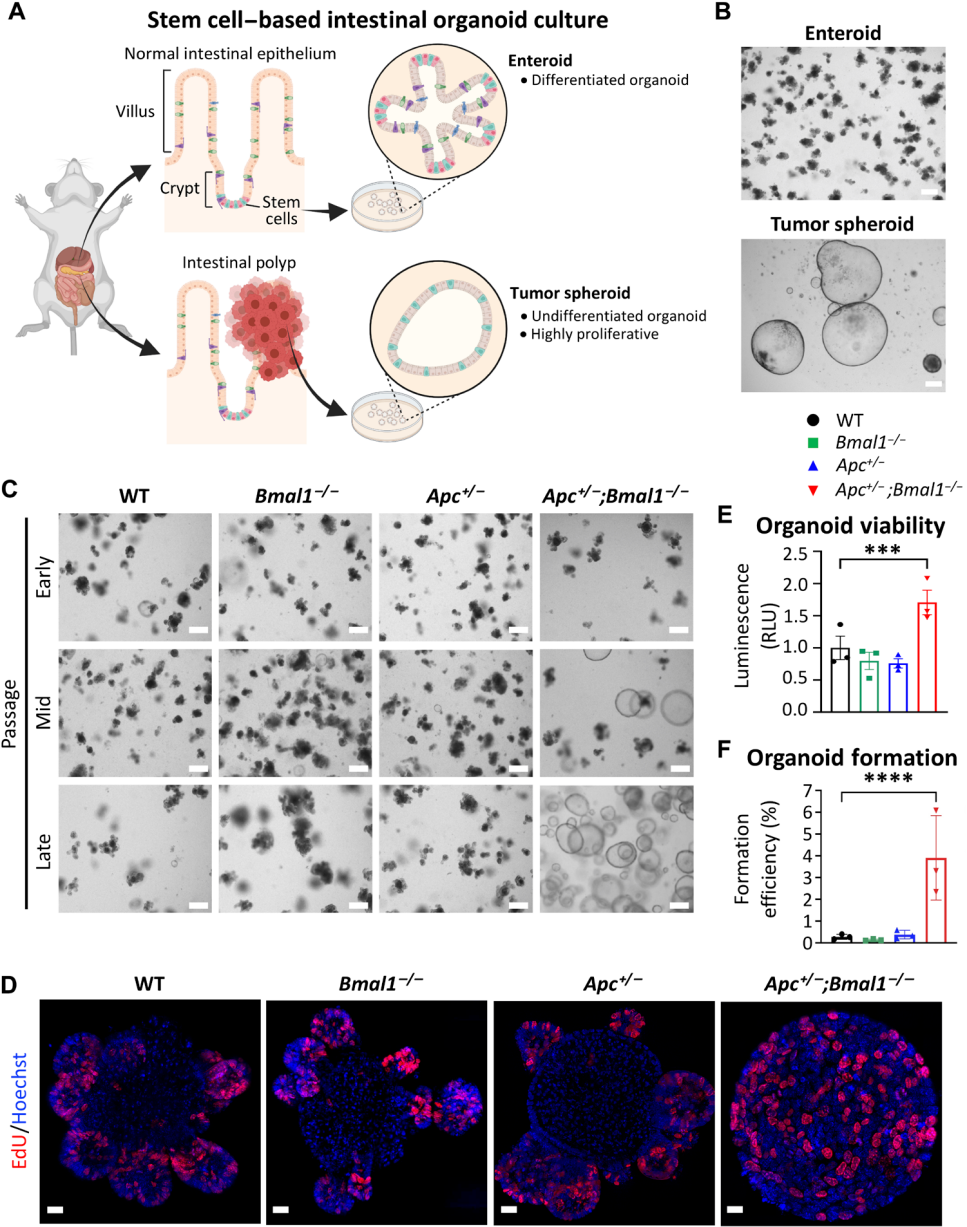
*Bmal1* disruption accelerates organoid spheroid formation and proliferation. (**A**) Schematic depicting stem cell–based intestinal organoid culture from normal intestinal epithelium and tumors. (**B**) Bright-field microscopy images of enteroid (top) and tumor spheroid (bottom) organoids grown in culture. Scale bars, 100 μm. (**C**) Bright-field microscopy images of organoids established from intestinal crypts at early, mid, and late passages. Scale bars, 100 μm. (**D**) EdU incorporation in organoids. Merged channels of EdU (red) and Hoechst (blue) were taken on a Zeiss Elyra 7 super-resolution confocal microscope. Scale bars, 20 μm. (**E**) Viability of organoids after 5 days of growth as determined using CellTiter-Glo 3D cell viability assay (*n* = 3 independent organoid lines per genotype). Luminescence was normalized to total protein amount to obtain relative light units (RLU). (**F**) Organoid formation of WT, *Bmal1*^−/−^, *Apc*^+/−^, and *Apc*^+/−^;*Bmal1*^−/−^ organoids after 5 days of culture. Organoid formation is shown as a percentage of single cells plated normalized to WT (*n* = 3 independent organoid lines per genotype). Data represent the mean ± SEM, and statistical significance was determined by one-way ANOVA with Tukey’s multiple comparison test. Asterisks represent *P* values from multiple comparisons, with ***P < 0.001 and ****P < 0.0001.

To dissect the molecular mechanism of how clock disruption impinges on epithelial cell growth properties, we adopted an ex vivo intestinal organoid model (Fig. 2, A and B). Mouse small intestinal crypts were isolated and allowed to form organoids, which can self-renew, proliferate, and differentiate into various IEC types (*57*). A total of four to six biological replicates of organoids were developed from independent mice and used for further experiments. Small intestinal organoids from WT, *Bmal1^−/−^,* and *Apc^+/−^* mice all formed the typical enteroid structure, which is characterized by stem cell–enriched budding crypts (Fig. 2, C and D, and fig. S3J). Organoids isolated from *Apc^+/−^;Bmal1^−/−^* mice initially grew as enteroids, yet over several consecutive passages, organoid morphology developed into tumor spheroids (Fig. 2C). We observed this transformation in culture from six independent *Apc^+/−^;Bmal1^−/−^* mice, while WT, *Bmal1^−/−^,* and *Apc^+/−^* organoids did not transform (Fig. 2C). Organoids collected before passage 10 were considered early passage, and transformed *Apc^+/−^;Bmal1^−/−^* organoids collected after 15 passages were considered late passage. Using 5-ethynyl-2′-deoxyuridine (EdU) incorporation and confocal microscopy, a profound proliferative capacity was observed in cells throughout the transformed *Apc^+/−^;Bmal1^−/−^* organoids, while EdU incorporation was localized to the enteroid bud in WT, *Bmal1^−/−^,* and *Apc^+/−^* organoids (Fig. 2D and fig. S3J). Using CellTiter-Glo to assess cell viability, we found that transformed *Apc^+/−^;Bmal1^−/−^* organoids demonstrated significantly increased viability versus all other genotypes (Fig. 2E). In addition, organoid formation efficiency was determined by calculating the ratio of mature organoids formed relative to the number of single cells seeded. A statistically significant increase in formation was observed from transformed *Apc^+/−^;Bmal1^−/−^* organoids, as compared to all other genotypes (Fig. 2F). These data indicate that synergistic *Bmal1* disruption and *Apc* loss result in a hyperproliferative stem-like state in intestinal organoids, suggesting an important role in accelerating transformation.

### *Bmal1* disruption rewires global gene expression of intestinal organoids

**Fig. 3.**
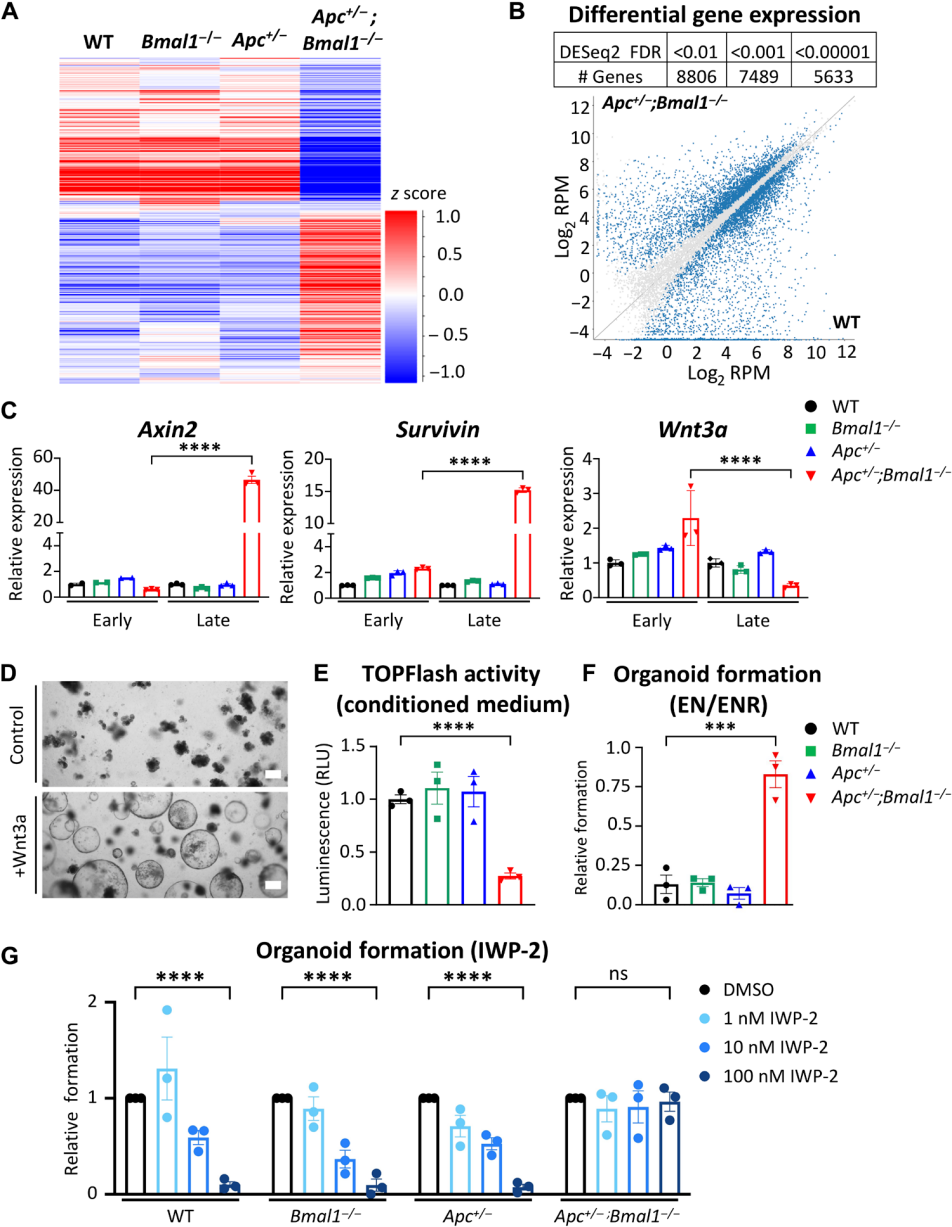
*Bmal1* disruption induces global transcriptome shift and deregulation of Wnt signaling in intestinal organoids. RNA-seq was performed using late-stage WT, *Bmal1*^−/−^, *Apc*^+/−^, and *Apc*^+/−^;*Bmal1*^−/−^ organoids (*n* = 3 independent organoid lines per genotype). (**A**) Heatmap depicting gene expression in organoids. (**B**) Table indicating the number of differentially expressed genes (DEGs) at different false discovery rate (FDR) thresholds and scatterplot showing expression of transcripts in *Apc*^+/−^;*Bmal1*^−/−^ compared to WT. Quantified reads are normalized per million mapped reads and log_2_-transformed (log_2_ RPM). DEGs with an FDR < 0.001 are highlighted in blue on the graph. (**C**) Expression of Wnt signaling genes *Axin2*, *Survivin*, and *Wnt3a* was determined by qPCR using WT, *Bmal1*^−/−^, *Apc*^+/−^, and *Apc*^+/−^;*Bmal1*^−/−^ organoids at early stage (before transformation of *Apc*^+/−^;*Bmal1*^−/−^ organoids) or late stage (after transformation of *Apc*^+/−^;*Bmal1*^−/−^ organoids). (**D**) Bright-field microscopy images of WT organoids treated with Wnt3a conditioned medium for 5 days. Scale bars, 100 μm. (**E**) TOPFlash reporter assay in human embryonic kidney (HEK) 293T cells treated with conditioned medium from WT, *Bmal1*^−/−^, *Apc*^+/−^, and *Apc*^+/−^;*Bmal1*^−/−^ organoids. Luciferase activity from each genotype is shown normalized to WT organoids, and RLU are shown (*n* = 3 independent organoid lines per genotype). (**F**) Relative organoid formation of WT, *Bmal1*^−/−^, *Apc*^+/−^, and *Apc*^+/−^;*Bmal1*^−/−^ organoids in R-Spondin–depleted medium (EN) normalized to formation in ENR medium (*n* = 3 independent organoid lines per genotype). (**G**) Organoid formation of WT, *Bmal1*^−/−^, *Apc*^+/−^, and *Apc*^+/−^;*Bmal1*^−/−^ organoids after treatment with increasing concentrations of inhibitor of Wnt signaling 2 (IWP-2). The formation at each concentration is presented as fold change relative to dimethyl sulfoxide (DMSO) vehicle treatment (*n* = 3 independent organoid lines per genotype). Data represent the means ± SEM. Statistical significance was determined by one-way ANOVA for (C), (E), and (F) and two-way ANOVA for (G) with Tukey’s multiple comparison test. Asterisks represent *P* values from multiple comparisons, with ***P < 0.001 and ****P < 0.0001. ns, not significant.

To define the putative role of *Bmal1* in tumor progression of the intestinal epithelium, we profiled gene expression using RNA sequencing (RNA-seq) from three independent biological replicates of late-passage WT, *Bmal1^−/−^*, *Apc^+/−^*, and *Apc^+/−^;Bmal1^−/−^* organoids. Assessment of the variance between each organoid line indicated that *Apc^+/−^;Bmal1^−/−^* organoids were distinctly different from the other three genotypes (fig. S4A). Differential gene expression analysis revealed profound global rewiring of transcription in *Apc^+/−^;Bmal1^−/−^* organoids relative to the other three genotypes (Fig. 3, A and B). Scatterplots showing the normalized read counts of *Bmal1^−/−^* or *Apc^+/−^* relative to WT revealed smaller contributions of single mutants versus double-mutant organoids on global gene expression (fig. S4, B and C). In addition, we observed large fold increases and decreases in gene expression between *Apc^+/−^;Bmal1^−/−^* and the other three genotypes (fig. S5A). Using Gene Set Enrichment Analysis (GSEA), we identified the up-regulation of relevant pathways involved in tumorigenesis, including terms associated with CRC, hypoxia-inducible factor 1, and vascular endothelial growth factor signaling, while terms such as transcriptional misregulation in cancer and fatty acid degradation were significantly suppressed (fig. S4D). Consistent with previous findings indicating that hyperactivation of the Wnt signaling pathway is involved in driving CRC (*2*–*4*), we observed significant enrichment of Wnt signaling in *Apc^+/−^;Bmal1^−/−^* organoids (fig. S4D). In addition, we observed a strong enrichment of proinflammatory terms including tumor necrosis factor, nuclear factor κB, interleukin-17, and Toll-like receptor signaling, suggesting a strong immune response in our model (fig. S4D). We also observed an epithelial-to-mesenchymal (EMT) signature with significant decreases in epithelial markers and a corresponding increase in mesenchymal gene signatures in *Apc^+/−^;Bmal1^−/−^* organoids. In addition, we saw increased expression of stemness markers and putative markers of colon cancer–initiating cells such as CD44 (*Cd44*) and CD133 (*Prom1*). This supports our previous findings that *Apc^+/−^;Bmal1^−/−^* organoids have a much greater proliferative capacity and enhanced stem-like state (Fig. 2, D to F). Last, we observed changes in DNA repair genes related to nonhomologous end joining (NHEJ) and homology-directed repair (HDR) (fig. S5B). Together, these data indicate that concurrent *Bmal1* and *Apc* loss significantly rewires global gene expression programs involved in driving intestinal tumor progression.

### Disruption of the intestinal clock activates ligand-independent Wnt signaling

It has been previously demonstrated that the circadian clock regulates Wnt signaling in normal intestine (*46*), and our RNA-seq analysis of *Apc^+/−^;Bmal1^−/−^* organoids identified Wnt signaling as a significantly enriched pathway (fig. S4D). The molecular mechanism of how disruption of *Bmal1* hyperactivates intestinal Wnt signaling in CRC is unknown. To address this question, we first profiled changes in gene expression in early-stage *Apc^+/−^;Bmal1^−/−^* enteroids versus late-stage tumor spheroids to determine whether progressive changes in Wnt signaling had occurred in culture. Notably, classical Wnt target genes *Axin2* and *Birc5* (also known as *Survivin*) were significantly up-regulated only in late-passage *Apc^+/−^;Bmal1^−/−^* organoids that grow as tumor spheroids (Fig. 3C). In addition, nuclear β-catenin localization is increased in late-passage *Apc^+/−^;Bmal1^−/−^* organoids compared to *Apc^+/−^* enteroids (fig. S6, A and B). To determine whether activated Wnt signaling is sufficient to drive the morphological switch from enteroid to spheroid state, WT organoids were treated with Wnt3a conditioned medium. Within 3 to 5 days, a shift from enteroid to spheroid morphology was visible in WT organoids in a Wnt3a dose-dependent manner (Fig. 3D and fig. S6C). These data suggest that Wnt signaling is likely responsible for the morphological shift to spheroids observed in our organoid model and that canonical Wnt/β-catenin signaling becomes hyperactivated in *Apc^+/−^;Bmal1^−/−^* organoids.

Wnt3a is a known ligand of TCF/LEF-dependent canonical Wnt signaling (*58*, *59*). Therefore, we analyzed *Wnt3a* gene expression and found that it was down-regulated in late-passage *Apc^+/−^;Bmal1^−/−^* organoids (Fig. 3C). In addition, activities of secreted Wnt ligands in conditioned medium collected from organoids, as determined by TOPFlash reporter assay (*11*), were significantly lower in late-passage *Apc^+/−^;Bmal1^−/−^*organoids (Fig. 3E). These data indicate that autocrine Wnt ligand activity is lower in *Apc^+/−^;Bmal1^−/−^* organoids. Thus, we hypothesized that these transformed spheroids proliferate in a Wnt ligand–independent manner. To test whether disruption of *Bmal1* and *Apc* results in Wnt ligand independence, organoid formation was determined in the absence of supplemented Wnt ligand. Organoid growth medium is supplemented with epidermal growth factor (EGF), noggin, and R-spondin (ENR). The R-Spondin family are known activators of Wnt signaling and can enhance the autocrine activity of Wnt ligands produced by the organoids (*60*). Therefore, we used EN medium (without R-Spondin) to define organoid formation in the absence of exogenous Wnt-like ligand. *Apc^+/−^;Bmal1^−/−^* organoids grown in EN medium had a formation efficiency similar to ENR, whereas WT and single-mutant organoids demonstrated a significantly reduced organoid formation capacity when deprived of Wnt-like ligands (Fig. 3F). The small-molecule inhibitor of Wnt signaling 2 (IWP-2) is a reported antagonist of Wnt signaling by blocking the processing and secretion of Wnt ligands (*61*), including Wnt3a (*62*). To determine whether organoids were Wnt ligand dependent, cultures were treated with increasing doses of IWP-2, and organoid formation efficiency was determined. While WT, *Bmal1^−/−^,* and *Apc^+/−^* organoids were sensitive to IWP-2 treatment in a dose-dependent manner, *Apc^+/−^;Bmal1^−/−^* organoids were resistant to IWP-2 inhibition and were able to efficiently form organoids at all doses tested (Fig. 3G). Consistent with our RNA-seq results, these data suggest that *Apc^+/−^;Bmal1^−/−^* organoids hyperactivate Wnt signaling in a ligand-independent manner, which could sustain enhanced viability, proliferation, and a stem-like state.

### *Bmal1* disruption accelerates *Apc* LOH

**Fig. 4.**
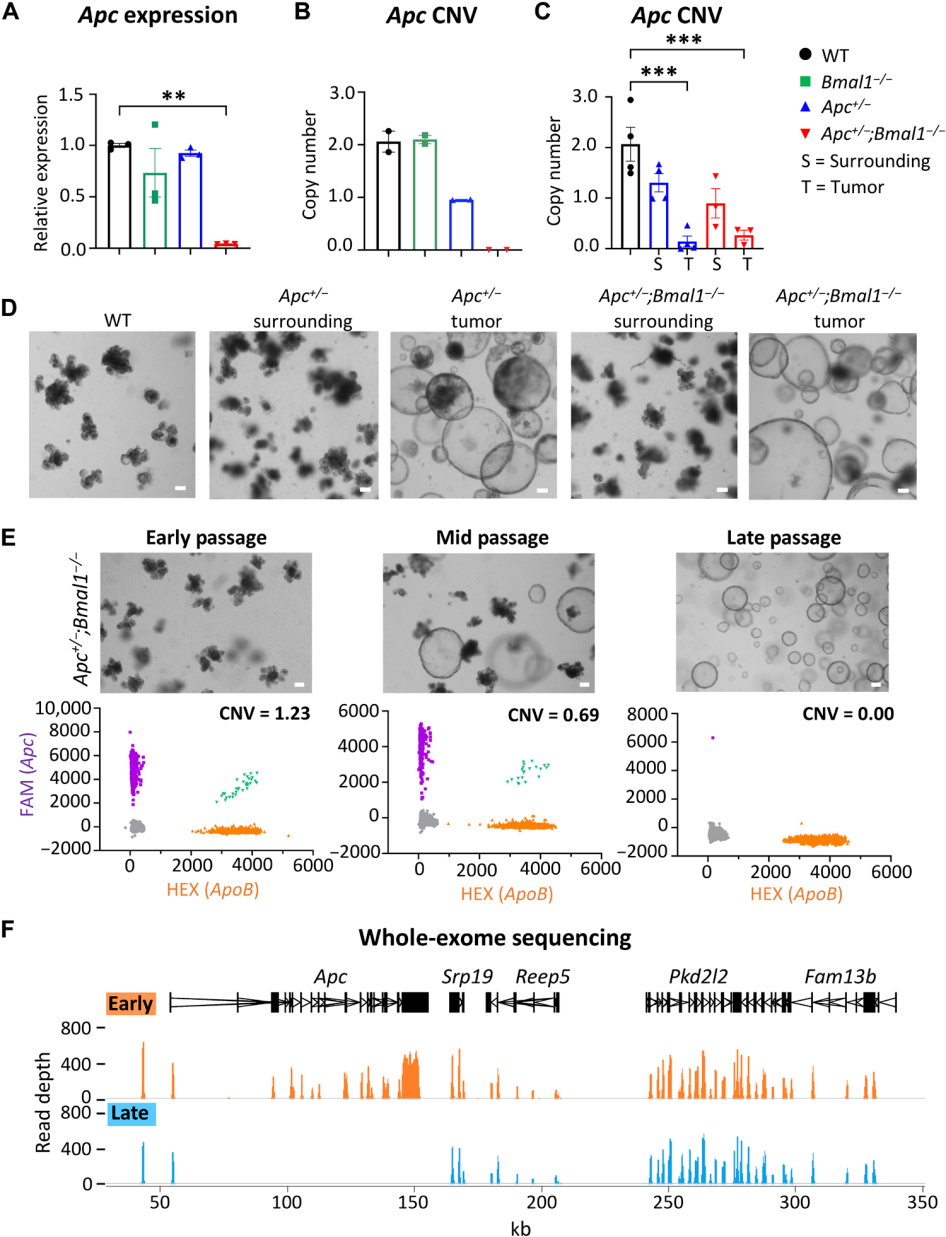
Intestinal clock disruption accelerates *Apc* LOH. (**A**) Quantification of *Apc* mRNA in late-stage organoids as determined by qPCR. (**B**) CNV was determined in WT, *Bmal1*^−/−^, *Apc*^+/−^, and *Apc*^+/−^;*Bmal1*^−/−^ organoids using dPCR (*n* = 2 independent organoid lines per genotype). (**C**) CNV was determined in WT organoids as well as tumor and surrounding *Apc*^+/−^ and *Apc*^+/−^;*Bmal1*^−/−^ organoids using dPCR (*n* = 3 to 4 independent organoid lines per genotype). (**D**) Microscope images of WT organoids along with tumor and surrounding *Apc*^+/−^ and *Apc*^+/−^;*Bmal1*^−/−^ organoids. Scale bars, 100 μm. (**E**) Microscope images of early-, mid-, and late-passage *Apc*^+/−^;*Bmal1*^−/−^ organoids along with dPCR scatterplots. FAM represents *Apc*-positive partitions, while HEX represents a reference genomic locus. Scale bars, 100 μm. (**F**) WES of gDNA from early- and late-passage *Apc*^+/−^;*Bmal1*^−/−^ organoids. Browser view shows exonic read depth at a region of chromosome 18, which includes *Apc.* Early (orange) and late (blue) peaks are the average quantification of reads in four independent organoid lines per condition. mRNA tracks are indicated in black, and the *x* axis indicates length in kilo–base pairs (kbp). Data represent the mean ± SEM. Statistical significance was determined by one-way ANOVA with Tukey’s multiple comparison test for (A) and (C). Asterisks represent *P* values from multiple comparisons, with **P < 0.01 and ***P < 0.001.

Although our GEMM is a heterozygous deletion of one allele of *Apc*, expression was unexpectedly lost in late-stage *Apc^+/−^;Bmal1^−/−^* spheroids, as determined by our RNA-seq analysis and qPCR (Fig. 4A). The *Apc* locus is a known target of point mutations, insertions, deletions, and LOH events, which have been described to drive tumor progression (*14*, *63*). In addition, the circadian clock is reported to be involved in the DNA damage response and DNA repair (*39*, *64*–*67*). Disruption of the clock may therefore accelerate mutagenic burden. We hypothesized that *Bmal1* disruption may drive transformation and tumor spheroid formation by increasing the mutation rate at the *Apc* locus and even genome-wide. To address this question, we first developed a dPCR assay to determine whether *Apc* copy number variation (CNV) could be the underlying cause of loss of gene expression. We designed a fluorescein amidite (FAM)-labeled probe to detect the exon 15 region of the *Apc* locus that is deleted in our *Apc^+/−^* models and compared that to a control genomic locus [Hexachlorofluorescein (HEX)-labeled probe] that is stable during cancer progression. This dPCR assay divides gDNA into 20,000 unique partitions, each containing a single PCR reaction to enable precise copy number quantification. We performed dPCR on gDNA isolated from organoids from each genotype. WT and *Bmal1^−/−^* organoids had an *Apc* copy number proximal to 2, while *Apc^+/−^* organoids had an expected copy proximal to 1 (Fig. 4B). Notably, the transformed *Apc^+/−^;Bmal1^−/−^* spheroids had an *Apc* copy number of 0 (Fig. 4B). We also established intestinal organoids from tumor and normal surrounding epithelium from our GEMM. We observed a loss of *Apc* copy number in *Apc^+/−^* and *Apc^+/−^;Bmal1^−/−^* tumor organoids versus WT (Fig. 4C). Similarly, these tumor-derived organoids immediately grew as spheroids (Fig. 4D), which resembled the transformed morphology of late-passage *Apc^+/−^;Bmal1^−/−^* organoids (Fig. 2, A to C). These data suggest that an LOH event occurred at the *Apc* genomic locus in *Apc^+/−^;Bmal1^−/−^* spheroids, explaining the complete loss of *Apc* gene expression (Fig. 4A). To define whether *Apc* copy number is lost during the morphological switch from enteroid to spheroid, gDNA was isolated from untransformed early-passage, mid-passage, and fully transformed late-passage *Apc^+/−^;Bmal1^−/−^* organoids. While the early-passage *Apc^+/−^;Bmal1^−/−^* organoids had an *Apc* copy number proximal to 1, this was reduced in the mid passage and reached 0 in the late-passage *Apc^+/−^;Bmal1^−/−^* organoids (Fig. 4E). These data illustrate that a loss of *Apc* copy number occurs as *Apc^+/−^;Bmal1^−/−^* organoids transform from normal enteroids to tumor spheroids.

To define the region of copy number loss within the *Apc* locus and identify additional genome-wide mutations, WES was performed using gDNA isolated from early- and late-passage *Apc^+/−^;Bmal1^−/−^* organoids. At the global level, similar read depths were observed between early and late passage (fig. S7A), indicating no major chromosomal abnormalities. WES confirmed that *Apc* LOH occurred in transformed late-passage *Apc^+/−^;Bmal1^−/−^* spheroids, as exonic reads were lost in nearly the entire coding region of the *Apc* locus (Fig. 4F). The observed difference in exonic reads was notably specific for *Apc*. Plotting fold changes in read count between early and late passage revealed a loss of nearly all *Apc* transcripts in late-passage organoids (fig. S7B). While this global analysis highlights the specificity for Apc LOH, it does not examine point mutations or insertions/deletions (indels) in key drivers of CRC that could be occurring genome-wide. Therefore, variant calling was performed on the WES data to map mutations. Although many indels and sporadic point mutations were observed genome-wide in late-passage *Apc^+/−^;Bmal1^−/−^* organoids, no consistent mutations were identified in each of the four organoid lines corresponding to driver genes of CRC progression, including *Kras*, *Nras*, *Braf*, *p53*, *Smad4*, *Pik3ca*, and *c-Myc* (fig. S7C). Together, these data implicate *Bmal1* as a safeguard in maintaining genome stability and *Apc* copy number, which are important in hyperactivating Wnt signaling and accelerating intestinal transformation.

### Intestinal clock disruption activates the MYC oncogene

We identified that loss of *Bmal1* and *Apc* resulted in hyperactivated Wnt signaling to potentially drive intestinal transformation. *c-Myc* is a well-known Wnt target gene (*51*) and plays an essential role in activating glycolysis and branch point pathways to sustain the heightened metabolic demand of rapidly proliferating cells (*68*, *69*). This Warburg effect is a hallmark feature of many tumors to provide essential building blocks to sustain cell growth (*70*). Furthermore, glutamine is a highly abundant circulating amino acid, and several cancer types display glutamine addiction (*71*–*73*). Glutaminolysis is the process by which cells can uptake glutamine and convert it to glutamate (Glu) and subsequently α-ketoglutarate (α-KG) to help fuel the tricarboxylic acid (TCA) cycle (*74*). In addition, *c-Myc* overexpression and amplification have been identified in human CRC (*75*–*77*), and these tumors exhibit features of heightened glycolytic metabolism (*78*, *79*).

**Fig. 5.**
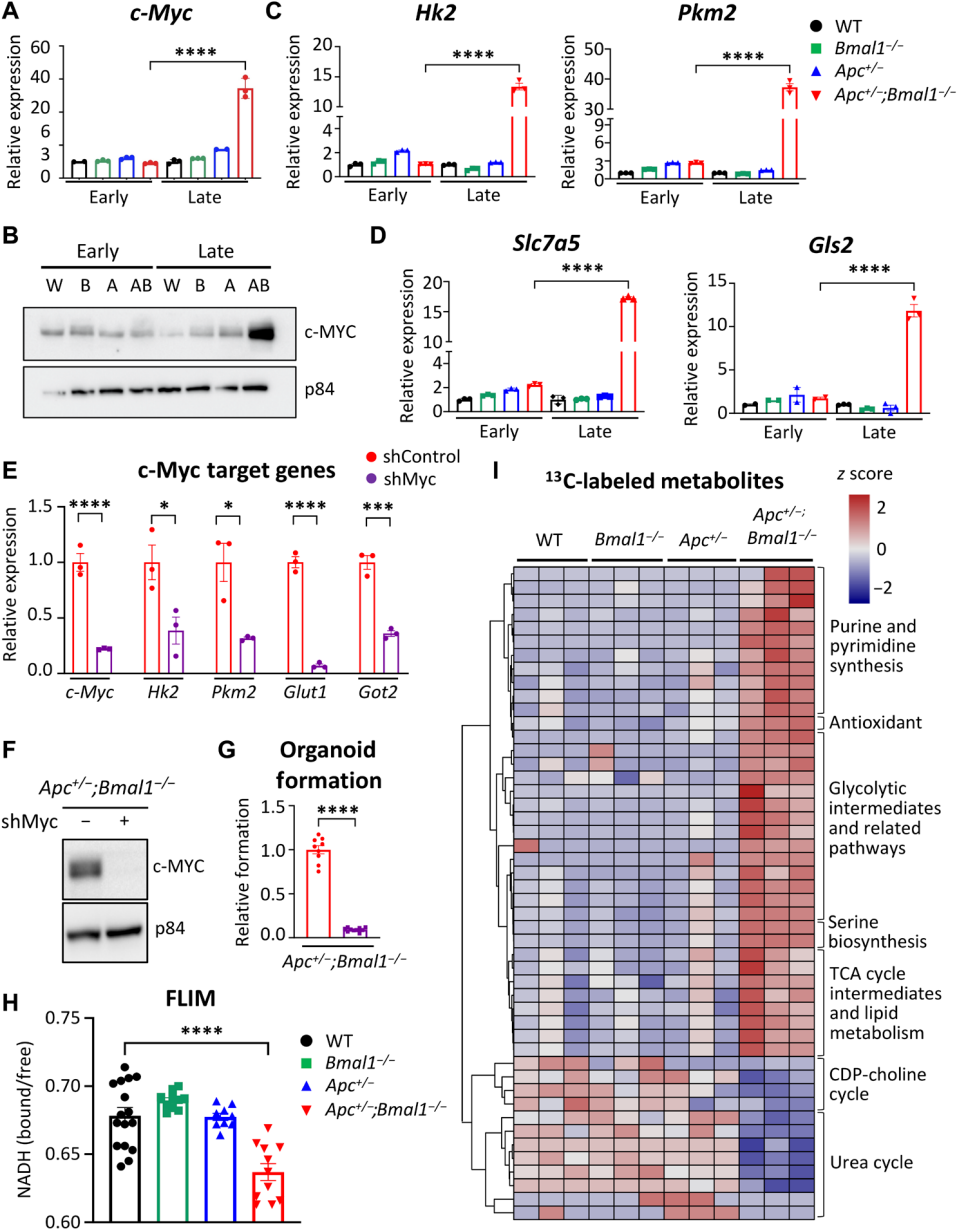
Increased MYC and glycolytic metabolism by disruption of the intestinal clock. (**A**) The expression of *c-Myc* in early- versus late-stage WT, *Bmal1*^−/−^, *Apc*^+/−^, and *Apc*^+/−^;*Bmal1*^−/−^ organoids, as determined using qPCR (*n* = 3 independent experiments). (**B**) Protein abundance of c-MYC shown by Western blot relative to p84 protein abundance in early- versus late-stage organoids. W, WT; B, *Bmal1*^−/−^; A, *Apc*^+/−^; AB, *Apc*^+/−^;*Bmal1*^−/−^ organoids. The expression of c-MYC target genes involved in (**C**) glycolysis and (**D**) glutaminolysis was determined using qPCR in WT, *Bmal1*^−/−^, *Apc*^+/−^, and *Apc*^+/−^;*Bmal1*^−/−^ organoids at early or late stages. (**E**) shRNA-mediated knockdown of *c-Myc* in *Apc*^+/−^;*Bmal1*^−/−^ organoids. Gene expression analysis of *c-Myc*, *Hk2*, *Pkm2*, *Glut1*, and *Got2* by qPCR (*n* = 3 independent experiments). (**F**) Knockdown of *c-Myc* protein abundance validated using Western blot and shown relative to p84 abundance. (**G**) Effect of *c-Myc* knockdown on organoid formation in *Apc*^+/−^;*Bmal1*^−/−^ organoids. Organoid formation is presented as fold change relative to the control infection (*n* = 8 independent experiments). (**H**) Ratio of free NADH (reduced form of nicotinamide adenine dinucleotide) versus bound NADH as measured by fluorescence lifetime imaging microscopy (FLIM) in WT, *Bmal1*^−/−^, *Apc*^+/−^, and *Apc*^+/−^;*Bmal1*^−/−^ organoids. Distribution of NADH signals was calculated by phasor analysis of segmented pixels. (**I**) Hierarchical clustered heatmap of significantly changed metabolites from ^13^C tracing metabolomics analysis in WT, *Bmal1*^−/−^, *Apc*^+/−^, and *Apc*^+/−^;*Bmal1*^−/−^ organoids (*n* = 3 independent organoid lines per genotype). Data represent the mean ± SEM. Statistical significance was determined by one-way ANOVA with Tukey’s multiple comparisons for (A), (C), and (D) and with Student’s unpaired *t* test for (E) and (G). Asterisks represent *P* values from *t* test or multiple comparisons, with *P < 0.05, ***P < 0.001, and ****P < 0.0001.

We identified in our RNA-seq analysis that expression of genes encoding many enzymes governing glycolysis and glutaminolysis were up-regulated in late-passage *Apc^+/−^;Bmal1^−/−^* organoids (fig. S5C). Therefore, we hypothesized that disruption of intestinal *Bmal1* drives *Apc* LOH and up-regulates Wnt-dependent *c-Myc* expression and subsequent activation of glycolytic metabolism. Using early-passage versus late-passage organoids, we determined that *c-Myc* expression is significantly up-regulated only in late-passage transformed *Apc^+/−^;Bmal1^−/−^* organoids (Fig. 5A). This increase in gene expression corresponded with an impressive accumulation of MYC protein abundance in late-passage *Apc^+/−^;Bmal1^−/−^* organoids (Fig. 5B). Next, we profiled the expression of MYC target genes involved in regulating glycolysis and glutaminolysis, which are known metabolic pathways activated by MYC in transformed cells (*80*, *81*). Expression of hexokinase 2 (*Hk2*), pyruvate kinase M2 (*Pkm2*), glucose transporter 1 (Glut1 or *Slc2a1*), and glutamic-oxaloacetic transaminase 2 (*Got2*) were significantly increased in transformed, late-stage *Apc^+/−^;Bmal1^−/−^* organoids (Fig. 5C and fig. S8A). Similarly, MYC target genes involved in glutaminolysis, such as glutamine transporter (*Slc7a5*) and glutaminase 2 (*Gls2*), were significantly up-regulated in late-stage *Apc^+/−^;Bmal1^−/−^* organoids (Fig. 5D). To validate these findings in our GEMM, we performed gene expression analysis using *Apc^+/−^* and *Apc^+/−^;Bmal1^−/−^* tumor-derived organoids. Our data demonstrate that *c-Myc* expression and MYC target genes are hyperactivated in organoids derived from tumor versus normal surrounding epithelium (fig. S6D). To define the causal role of MYC in regulating stem-like features and proliferation of *Apc^+/−^;Bmal1^−/−^* organoids, we performed knockdown of *c-Myc* using a short hairpin RNA (shRNA) silencing approach. *c-Myc* knockdown effectively reduced gene expression and protein abundance in two independent organoid lines (Fig. 5, E and F, and fig. S8, B and C) and significantly dampened MYC target gene expression (Fig. 5E and fig. S8B). MYC knockdown also significantly inhibited the formation of *Apc^+/−^;Bmal1^−/−^* organoids (Fig. 5G and fig. S8D). In addition, confocal images of EdU incorporation illustrate that knockdown of *c-Myc* decreases *Apc^+/−^;Bmal1^−/−^* organoid size and proliferation (fig. S8, E and G). These results suggest that MYC activation is essential for enhanced stemness and growth of transformed *Apc*^+/−^;*Bmal*1^−/−^ organoids.

### MYC activates increased glycolytic metabolism to sustain organoid proliferation

To determine whether *Bmal1* and *Apc* disruption altered the metabolism of organoids and caused a shift toward heightened glycolytic metabolism, we first used the phasor approach to fluorescence lifetime imaging microscopy (FLIM). FLIM has been previously used to map cellular metabolism in the intestine (*82*, *83*). FLIM monitors dynamic shifts in patterns of aerobic glycolysis versus mitochondrial oxidative metabolism by following the fluorescence decay of NADH (reduced form of nicotinamide adenine dinucleotide) (*84*). Free and unbound NADH is indicative of active glycolysis, while bound NADH is representative of oxidative phosphorylation. Using intestinal organoids from all genotypes, we observed notable shifts toward enhanced glycolytic metabolism in the late-passage *Apc^+/−^;Bmal1^−/−^* organoids, pseudo-colored as pink spots, compared to WT and single mutants (fig. S8H). This shift was associated with a corresponding significant decrease in oxidative phosphorylation relative to glycolysis in double-mutant organoids (Fig. 5H).

To demonstrate that *Bmal1* disruption activates glycolytic metabolism, we used stable isotope tracing coupled with mass spectrometry (MS). Metabolic tracing using [U-^13^C]glucose revealed that glycolytic metabolites and lactate were highly labeled in late-passage *Apc^+/−^;Bmal1^−/−^* organoids (Fig. 5I and fig. S9A). In addition, biosynthetic pathways that branch off of glycolysis such as the hexosamine and serine synthesis pathways were abundantly labeled in *Apc^+/−^;Bmal1^−/−^* organoids (Fig. 5I and fig. S9A). Oncogenic MYC promotes anabolic reactions that support cell proliferation and growth. Consistent with this, we observed an increase in labeled purine and pyrimidine derivatives and a reduction of labeled intermediates of the urea cycle and uric acid in double-mutant transformed organoids (Fig. 5I and fig. S9A). These data suggest that increased generation of building blocks are needed to support accelerated cell growth in *Apc^+/−^;Bmal1^−/−^* organoids, as well as reduced catabolism of nitrogen sources that are needed for nucleotide synthesis to sustain DNA replication and adenosine triphosphate (ATP) production. In addition, MYC promotes mitochondrial metabolism, and several TCA cycle intermediates were significantly labeled in *Apc^+/−^;Bmal1^−/−^* organoids, including acetyl coenzyme A (Ac-CoA), citrate, succinyl-CoA, and malate (Fig. 5I and fig. S9A). In addition, labeled Glu and α-KG were more abundant in *Apc^+/−^;Bmal1^−/−^* organoids (fig. S9A), consistent with increased expression of genes involved in glutaminolysis (Fig. 5D). Aside from stable isotope tracing with labeled glucose, we also performed untargeted metabolomics in our organoid lines. An overall decrease in amino acid levels was observed in *Apc^+/−^;Bmal1^−/−^* organoids, which might reflect their increased incorporation into newly synthesized proteins (fig. S9B).

To confirm an essential role of glycolysis in growth and stemness of transformed *Apc^+/−^;Bmal1^−/−^* organoids, we used a nonmetabolizable glucose analog [2-deoxy-d-glucose (2DG)] or a GLUT1 inhibitor (WZB-117) to block the uptake of glucose. Both 2DG and WZB-117 showed significant dose-dependent reduction in organoid formation efficiency, suggesting that stem-like features of transformed *Apc^+/−^;Bmal1^−/−^* organoids are highly dependent on hyperactivated glycolytic metabolism (fig. S9, C and E). Notably, glucose uptake was measured using conditioned organoid medium, and we found an expected decreased consumption of glucose especially in the presence of 2DG (fig. S9, D and F). Together, FLIM data and metabolomics analyses illustrate that transformed *Apc^+/−^;Bmal1^−/−^* organoids exhibit heightened glycolytic metabolism and activation of branch point pathways that are required to sustain enhanced growth of cancer cells.

### Patient tumor samples exhibit dampened circadian rhythms

**Fig. 6.**
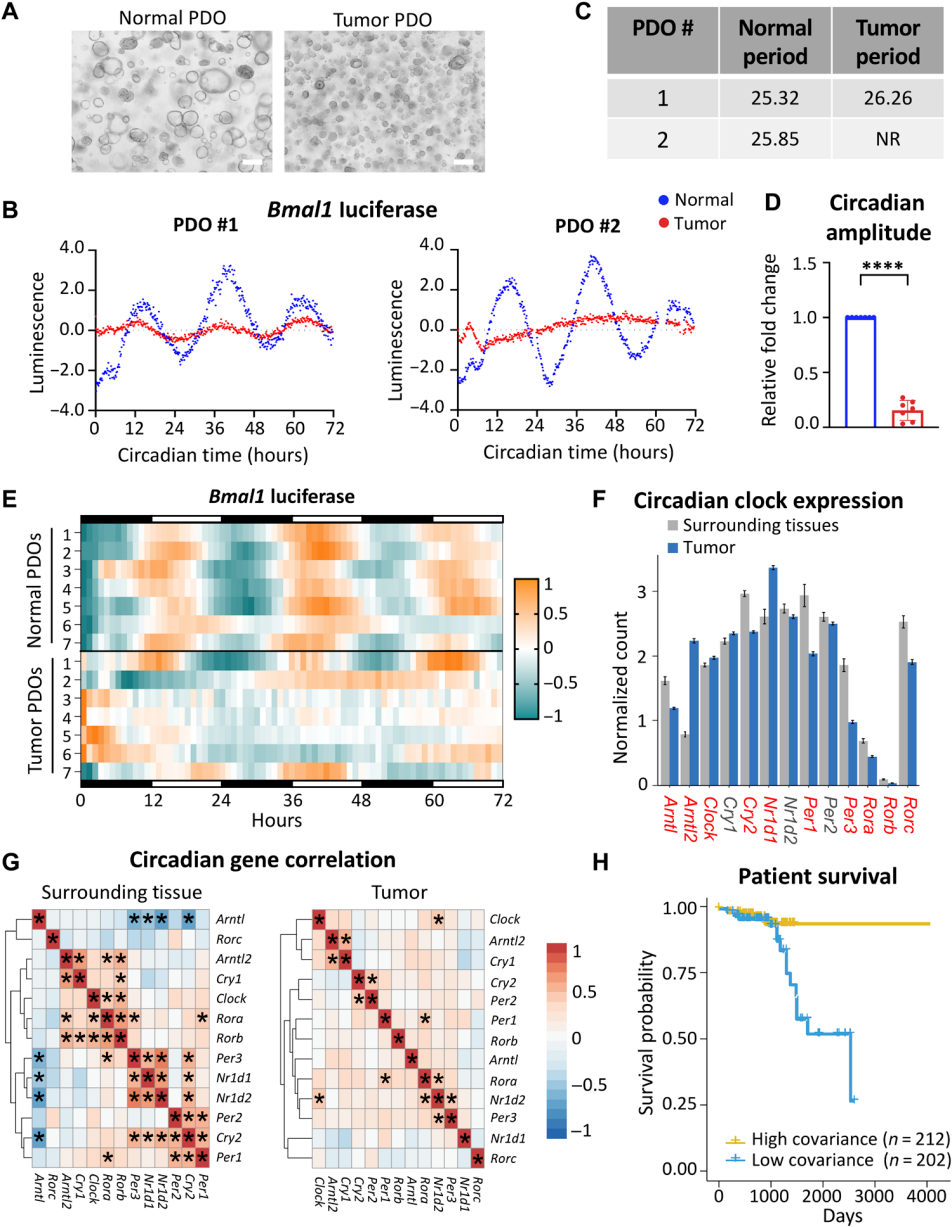
The circadian clock is disrupted in human CRC. (**A**) Representative bright-field microscopy images of organoids established from normal and tumor tissue from the same patient. Scale bars, 100 μm. (**B**) Detrended *Bmal1-*driven luminescence over time for matched normal and tumor PDOs. (**C**) Circadian period from PDOs was calculated in matched normal and tumor intestinal organoids using BioDare2. NR, nonrhythmic. (**D**) Circadian amplitude for each tumor-derived PDO normalized to the matched normal PDO from each of seven pairs. Amplitude was calculated using BioDare2. Data represent the mean ± SEM, and statistical significance was determined using Wilcoxon’s matched-pair signed-rank test. Asterisks represent the *P* value, with ****P < 0.0001. (**E**) Circadian rhythmicity determined by *Bmal1*-luciferase reporter activity of matched PDO pairs over 72 hours presented as a heatmap. Each row represents a normal or tumor PDO line (*n* = 7 independent PDO pairs). (**F**) Core clock gene expression in normal surrounding tissue (*n* = 57) versus colorectal tumors (*n* = 470) using TCGA. Data represent the mean ± SEM, and comparisons with a *P* value lower than 0.001 (two-way Student’s *t* test) are labeled red. (**G**) Heatmaps showing the correlation of gene expression among core clock genes in normal surrounding tissue and colorectal tumors. Asterisks represent significant covariance, where *P* < 0.001. (**H**) Patients with CRC in TCGA were binned into high-covariance (*n* = 212) or low-covariance (*n* = 202) categories on the basis of variance between core clock and Wnt pathway genes and shown relative to overall survival probability. *P* value was estimated using the log-rank method from Kaplan-Meier curves, *P* = 0.0032.

Our data suggest that *Bmal1* plays an important role in intestinal transformation in our GEMM, yet the role of the circadian clock in maintaining rhythms in the human intestine and how this is deregulated in CRC remain undefined. Therefore, we surveyed circadian rhythms in human tissue from excised normal colon versus colonic tumors. PDOs were established from these matched normal and tumor tissue samples taken during surgical resection (Fig. 6A and fig. S10, A and B). PDOs were then transduced with a *Bmal1*-driven luciferase reporter and synchronized with dexamethasone, and real-time bioluminescence was measured over 3 days. PDOs established from normal colon tissue displayed a robust circadian rhythm (Fig. 6, B to D and fig. S10C). Conversely, PDOs established from adjacent colonic tumors were arrhythmic or displayed an abnormal period, with significantly decreased circadian amplitude (Fig. 6, B to D and fig. S10C). Similar results were observed in paired PDOs from seven independent patients that were examined (Fig. 6E and fig. S10A). In addition, we surveyed the status of APC and abundance of BMAL1 protein in PDOs. APC showed the expected truncated and nonfunctional forms of the protein, and BMAL1 levels were decreased in tumor versus normal PDOs (fig. S10D). These results suggest that normal colon tissue maintains a functional circadian clock, while the clock is dampened or lost during tumorigenesis in patients with CRC.

To define the role of the circadian clock in larger patient populations, we determined the degree of conservation for genes and pathways identified in our mouse model within the context of human variation. We surveyed publicly available RNA-seq and phenotypic data from tumors and surrounding tissue in 512 patients with CRC in TCGA. Initial analyses showed that expression of 10 of 12 core clock genes was significantly altered between tumor and normal surrounding tissue (Fig. 6F). Moreover, while bidirectional correlations among the core clock gene network were significant and apparent within the surrounding normal tissue, these relationships were abolished within tumor samples of the same patients (Fig. 6G). These data suggest that the core clock is disrupted in human colorectal tumors. Consistent with the interaction between Wnt signaling and circadian genes playing a key role in the progression of tumorigenesis, stronger correlation significance was observed between the two pathways in the surrounding tissue compared to tumors (fig. S10E). To test whether interaction between these two pathways could be relevant for patient outcomes, individuals within TCGA were assigned a personalized score based on principal component contributions to gene variation in core clock and Wnt pathways (*85*–*87*). Specifically, individuals with high covariance in Wnt circadian genes were binned separately from those with low covariance. Those with low covariance in gene expression patterns, when clock and Wnt do not correlate, were found to have poor overall survival outcomes (Fig. 6H). Collectively, these data demonstrate that a functional clock is lost in human colorectal tumors, and population-level data further support a cross-talk between core clock and Wnt pathways in CRC progression and patient survival.

## DISCUSSION

**Fig. 7.**
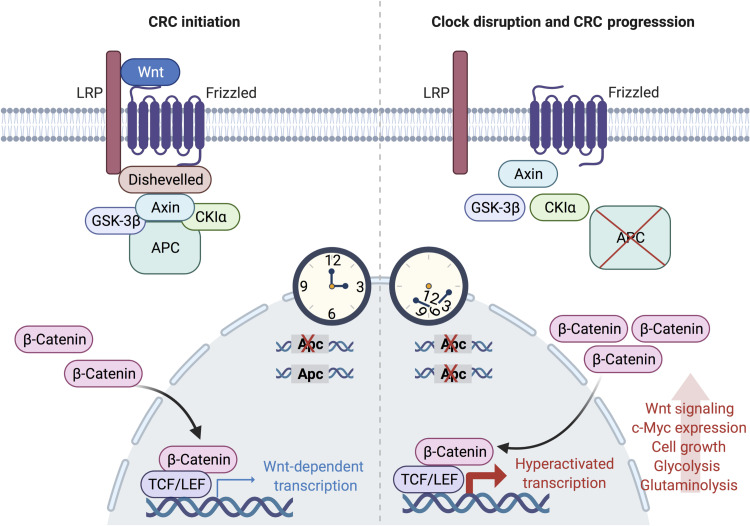
Model depicting how circadian clock disruption drives CRC progression. Disruption of *Bmal1* in the intestine accelerates *Apc* LOH, which results in the hyperactivation of Wnt signaling, including the Wnt-target gene *c-Myc*. In turn, MYC activates glycolytic metabolism and branch point pathways involved in supporting the demand of hyperproliferative cells. Together, circadian clock disruption drives proliferation and rewires metabolism of IECs to accelerate CRC progression in an *Apc* mutant background. Figure was created using biorender.com.

Our findings collectively indicate that genetic and environmental disruption of intestinal *Bmal1*, in the presence of *Apc* mutation, accelerates CRC progression in our newly developed GEMM. We identified an unexpected role for the clock in maintaining copy number fidelity at the *Apc* locus. Using an ex vivo intestinal organoid model, we demonstrate that *Apc^+/−^;Bmal1^−/−^* organoids transform by accelerating *Apc* LOH, which hyperactivates Wnt signaling and subsequently activates proliferative and metabolic pathways driven by c-Myc (Fig. 7).

The notable *Apc* LOH that occurs in the *Apc^+/−^;Bmal1^−/−^* double-mutant organoids indicates that a functional clock is required to maintain genome stability. While qPCR data show a loss of *Apc* expression, dPCR and WES demonstrate a loss of chromosomal DNA at the *Apc* locus, ruling out silencing of the intact *Apc* gene and indicating instability at the DNA level. Complete deletion of a portion of the chromosome requires a DNA double-strand break (DSB) that would necessitate repair through either NHEJ or HDR (*88*). To uncover which pathway is involved in the aberrant DSB repair resulting in *Apc* LOH, we further examined our RNA-seq data for changes in expression of DNA repair pathway components in the *Apc^+/−^;Bmal1^−/−^* double-mutant organoids (fig. S5B). Notably, we found a significant up-regulation in genes that would favor DNA end resection over DNA end protection, thus favoring HDR. While the large deletion at the intact *Apc* locus coupled with an increase in end resection factors strongly suggests that DSB repair is occurring through the deletion-prone pathways of either single-strand annealing or alternative DNA end-joining, future studies will focus on elucidation of the full LOH mechanism.

In the context of the normal intestinal epithelium, an intriguing cross-regulation has been reported, linking Wnt signaling and the circadian clock. Expression of the circadian clock components is reported in intestinal stem and progenitor cells, as well as certain differentiated cells of the intestine (*89*). In addition, the molecular clock is reported to regulate the cell cycle of intestinal stem cells after chemical damage induced by dextran sodium sulfate (*47*). These findings raise the question regarding the role of the core clock machinery in the different cell types of the intestine. In support of this concept, Wnt secretion from Paneth cells was reported to couple the circadian clock and the cell cycle in normal intestine (*46*). Collectively, these studies suggest that clock regulation could be cell type specific in the intestine. Moreover, multiple upstream regulatory cues could impinge on the intestinal clock. Previous findings have shown that the circadian clock is functional in intestinal stem cells and differentiated enterocytes and that Wnt and Hippo signaling converge to regulate clock-controlled stem cell function (*48*). In the context of tumorigenesis, these upstream signaling pathways that regulate clock function could differ, and this concept requires further exploration. Although several reports previously implicate the role of the clock in normal intestinal function, the molecular mechanism of how the circadian clock impinges on Wnt signaling in the context of CRC remained undefined. Our study now sheds light on the role of the circadian clock in regulating copy number integrity and how *Apc* LOH can affect Wnt signaling and MYC activation to accelerate CRC.

A cross-talk between the circadian clock and MYC-dependent signaling has been previously reported, yet how these two transcriptional networks operate and regulate one another has resulted in various hypotheses regarding mechanism of action. Published data clearly indicate that a bidirectional cross-talk exists where activated MYC can repress molecular clock components, and the clock can feedback and repress MYC signaling. For instance, MYC can activate the expression of *Rev-Erbα* and *Rev-Erbβ*, which can feedback and suppress *Bmal1* transcription (*41*). MYC has also been reported to work cooperatively with MIZ1 to repress clock gene expression (*90*). These findings also support the concept that CLOCK:BMAL1 and MYC regulate gene expression through common E-Box motifs that could implicate transcriptional competition as an important mechanism (*41*, *90*, *91*). Circadian repressors also play an important role in regulating the clock/MYC cross-talk. MYC can control the nuclear/cytoplasmic localization of the circadian repressors Period 1 (PER1) and PER2 (*92*) to potentially feedback and regulate clock-dependent transcription. Cryptochrome 2 (CRY2) has been reported to regulate the stability of MYC through the ubiquitin proteosome SCF^FBXL3^ complex (*40*), and CRY1 and CRY2 also modulate the expression of *c-Myc* (*93*). This regulation between the clock and MYC suggests that time of day potentially plays a key role in protein accumulation of these pathways, implicating stoichiometry of these transcription factors as a critical component of this cross-talk. Here, we reveal a previously unidentified mechanism of action that *Bmal1* loss drives *Apc* LOH and hyperactivation of Wnt signaling, which up-regulates *c-Myc* expression in IECs. Our findings provide an additional layer of complexity between the circadian clock and the cross-talk with MYC signaling, suggesting that tissue specificity and cell type are likely imperative in defining the mechanism of action. Stem cells and cancer-initiating cells are highly dependent on Wnt signaling, and therefore, our findings suggest that the bidirectional regulation of the clock and MYC could differ between stem cells and differentiated cells. Furthermore, we identified an EMT gene expression signature with an increase in stemness markers, suggesting that interactions between the clock and MYC could be involved in the progression of CRC by regulating the population of cancer-initiating cells.

Data from our GEMM indicate that genetic disruption of the circadian clock drives CRC progression, which is supported by substantial rewiring of cellular metabolism. However, the role of environmental disruption of the circadian clock in the context of CRC remains undefined. High-fat diet (HFD) is known to reprogram circadian metabolic rhythms (*94*–*96*). In addition, HFD and high-fructose diet are known to accelerate CRC by regulating de novo lipogenesis (*97*). HFD enhances the stemness of intestinal stem cells to drive tumorigenicity through peroxisome proliferator–activated receptor (PPAR)– and farnesoid X receptor–dependent mechanisms that potentially govern Wnt signaling (*98*, *99*). Given the role of the clock in regulating PPAR transcriptional pathways that control lipid metabolism under HFD (*94*, *100*), the mechanism of how HFD controls circadian pathways that can impinge on Wnt signaling, stemness, and intestinal stem cell function requires further exploration. Last, the potential for ameliorating the negative effects of HFD on circadian metabolism have been shown in the context of time-restricted feeding (TRF) (*101*, *102*), and how TRF could impinge on CRC prevention is an important future avenue of investigation. Collectively, our data establish the circadian clock as a key regulator of *Apc* copy number integrity that governs intestinal Wnt/β-catenin signaling during the progression of CRC. Our molecular findings suggest that environmental clock disruption through behavior and diet likely have important implications for young onset CRC.

## METHODS

### Mice

*Apc* mice that harbor a heterozygous floxed allele of exons 1 to 15 (*Apc*^*+/*Δ*ex1–15*^) (*19*) were crossed with *Bmal1* conditional mice that carry homozygous floxed alleles of exon 8 (*Bmal1*^fl/fl^) (*54*). Intestine-specific targeting of epithelial cells was achieved by crossing these conditional mice with Villin-Cre animals to create *Apc*^*+/*Δ*ex1–15*^;*Bmal1^fl/fl^;Villin-Cre* mice. Mice were purchased from the Jackson Laboratory (Bar Harbor, ME). All experiments were performed in accordance with the Institutional Animal Care and Use Committee (IACUC) guidelines at the University of California, Irvine. Animals were housed on a standard 12-hour light/12-hour dark paradigm and fed ad libitum. SD was performed on mice for 10 weeks by advancing the light phase by 8 hours every other day.

### Indirect calorimetry and MRI

Oxygen consumption, carbon dioxide release, RER, locomotor activity (counts), and food intake were monitored for individually housed mice using PhenoMaster metabolic cages (TSE Systems). Animals were entrained for 1 day in the metabolic cages before the start of each experiment to allow for environmental acclimation. Data were collected at 39-min intervals, and each cage was recorded for 3.25 min before time point collection. The light/dark schedule in the metabolic cages was maintained identical to the home cage environment. Body composition was measured using the EchoMRI Whole Body Composition Analyzer (Houston, TX).

### H&E staining and IHC

The ileum and colon were dissected, flushed with phosphate-buffered saline (PBS), and linearized longitudinally. The tissue was incubated in modified Bouin’s fixative (50% ethanol and 5% acetic acid) for 10 to 15 s, Swiss-rolled, and fixed overnight at room temperature in 4% paraformaldehyde (PFA). Paraffin embedding, tissue sectioning, and H&E staining were performed by the Experimental Tissue Resource (ETR). For IHC, formalin-fixed paraffin-embedded slides were incubated at 60°C for 1 hour. After deparaffinization and rehydration, antigen retrieval and peroxidase inactivation were carried out. Tissue sections were blocked with goat serum, avidin, and biotin (Vector Laboratories) and then incubated overnight with 1:100 anti–β-catenin (Cell Signaling Technologies, 8480). Slides were incubated with 1:200 biotinylated goat anti-rabbit immunoglobulin G (IgG) (Vector Laboratories), avidin biotin complex (ABC) solution (Vector Laboratories), and DAB Quanto (Thermo Fisher Scientific) and were counterstained with hematoxylin.

### IEC isolation and mouse model validation

For isolation of IECs, the ileum was harvested and opened longitudinally. Intestinal segments were vigorously shaken with Hanks’ balanced salt solution (HBSS) supplemented with 15 mM Hepes and 1× penicillin/streptomycin (P/S) and collected by centrifugation. Washed tissues were incubated in dissociation buffer [15 mM Hepes, 10 mM EDTA, 5% fetal bovine serum (FBS), and 1× P/S in HBSS] for 10 min at 37°C with vigorous shaking. After incubation, the remaining tissues were discarded, and IECs were collected by centrifugation. For validation of *Apc*, *Bmal1*, and *Villin-Cre* in IECs, isolated gDNA was used for quantitative real-time PCR using PowerUp SYBR Green Master Mix (Applied Biosystems) and was normalized to *Per1*. Primer sequences used for qPCR are listed in table S1.

### Mouse intestinal crypt isolation and organoid culture

The following protocol was used with minor modifications, based on previously published methods (*103*). The intestinal segment was dissected into small pieces and incubated in dissociation solution (PBS supplemented with 2 mM EDTA and 10 μM Rho kinase inhibitor Y-27632) for 1 hour at 4°C with agitation. Next, intestinal tissue pieces were shaken, strained, and centrifuged; pellets were resuspended in Matrigel (Corning Inc.); and ENR medium was added. ENR is a basal medium supplemented with recombinant murine EGF (50 ng/ml) (PeproTech), recombinant murine Noggin (50 ng/ml) (PeproTech), 1 mM *N*-acetylcysteine (Sigma-Aldrich), and 20% (v/v) of R-Spondin–conditioned medium (Cultrex *Rspo1*-expressing cells, Trevigen). The basal medium is advanced Dulbecco’s modified Eagle’s medium (DMEM)/F12 (Gibco) supplemented with 3 mM l-glutamine (Thermo Fisher Scientific), Primocin (100 μg/ml) (InvivoGen), and 10 mM Hepes (Sigma-Aldrich). For tumor-derived organoids, dissected polyps were prepared as described above, with an additional 1-hour incubation in digestion buffer [2.5% FBS, P/S (1 μg/ml), type IV collagenase (200 U/ml), and type II dispase in DMEM (125 μg/ml)].

### Organoid formation assay

Organoids were collected using Cell Recovery Solution (Corning) and were dissociated by vigorous resuspension in TrypLE Express (Thermo Fisher Scientific). After a 10-min incubation at 37°C, dissociated single cells were collected by centrifugation and resuspended in Matrigel, and ENR medium supplemented with 10 μM Y-27632 was added. Organoid formation efficiency was calculated by dividing the number of organoids formed by the total number of single cells plated after 5 days. For inhibitor experiments, ENR was supplemented 1 day after plating with IWP-2 (Thermo Fisher Scientific), 2DG (Sigma-Aldrich), or WZB-117 (Sigma-Aldrich). For the EN condition, formation assays were prepared in medium without R-Spondin.

### TOPFlash luciferase reporter assay

To make conditioned medium, organoids were provided with EN medium and collected after 48 hours. Human embryonic kidney (HEK) 293T cells were transfected with TOPFlash reporter (Trevigen) using BioT transfection reagent (Bioland Scientific) according to the manufacturer’s protocol. LacZ was ectopically expressed with the TOPFlash reporter as an internal control for transfection. Transfected HEK293T cells were incubated with conditioned medium from different organoid lines for 24 hours and harvested using luciferase lysis buffer [25 mM tris (pH 7.8), 2 mM EDTA, 1 mM dithiothreitol (DTT), 10% glycerol, and 1% Triton X-100]. For the luciferase assay, lysates were mixed with luciferase reaction buffer [20 mM tris (pH 7.8), 1.07 mM MgCl_2_, 2.7 mM MgSO_4_, 0.1 mM EDTA, 33.3 mM DTT, 470 μM beetle luciferin, 530 μM ATP, and 270 μM CoA], and luminescence was detected using a Varioskan LUX microplate reader (Thermo Fisher Scientific). β-Galactosidase activity was measured by mixing lysates with β-galactosidase assay buffer [60 mM Na_2_HPO_4_, 40 mM NaH_2_PO_4_, 10 mM KCl, 1 mM MgSO_4_, 50 mM β-mercaptoethanol, and *ortho*-nitrophenyl-β-galactoside (0.7 mg/ml)], and absorbance at 450 nm was measured. The total organoid protein amount was used for normalization.

### Glucose uptake assay

*Apc*^+/−^;*Bmal1*^−/−^ organoids were grown for 4 days in ENR medium and subsequently replaced with 2DG or WZB-117. After a 24-hour incubation, conditioned medium was collected and measured using a glucose (HK) assay kit (Sigma-Aldrich). Glucose consumption was calculated on the basis of the difference in glucose concentration between ENR and conditioned medium. Results were normalized to total protein content.

### Organoid immunofluorescence

Organoids were fixed with 4% PFA for 20 min. A permeabilization solution (0.5% Triton X-100 in PBS) was added to each well for 20 min. Organoids were blocked and then incubated with 1:75 β-catenin overnight at 4°C. Organoids were incubated with 1:600 goat anti-rabbit IgG cross-adsorbed Alexa Fluor 488 (Thermo Fisher Scientific, A11008) for 1 hour. To visualize nuclei, wells were incubated with 1× Hoechst 33342 (Thermo Fisher Scientific). The slides were imaged on a Zeiss LSM900 microscope. Nuclear β-catenin was quantified using Imaris version 9.0.0.

### EdU incorporation and staining

EdU was added to the organoids for 4 hours, followed by fixation in 4% PFA. For the EdU Click-iT reaction, organoids were treated with Click-iT cocktail (Thermo Fisher Scientific) and incubated for 30 min at room temperature. Organoids were stained with 1× Hoechst 33342 (Thermo Fisher Scientific) for 30 min. The slides were imaged on a Zeiss Elyra 7 superresolution microscope. Fifty to 100 *z*-stacks were imaged over 6 to 16 tiles. For processing, the *z*-stacks were first combined using a structured illumination microscope and then the tiles were stitched together.

### RNA extraction and gene expression analysis

Total RNA from organoids was extracted by using a Direct-zol RNA microprep kit (Zymo Research). To generate cDNA, equal amounts of total RNA from organoids were incubated with Maxima H Minus cDNA Synthesis Master Mix (Thermo Fisher Scientific) according to the manufacturer’s instructions. cDNA was used for quantitative real-time PCR using PowerUp SYBR Green Master Mix (Applied Biosystems). Gene expression was normalized to 18*S* ribosomal RNA. Primer sequences used for gene expression analysis by qPCR are listed in table S1.

### RNA-seq analysis

Polyadenylate enriched total RNA was used for library preparation, and paired-end 150–base pair (bp) sequencing was performed by Novogene. Raw reads were aligned to the current mouse genome build (GRCm39) using STAR aligner (*104*). PCR duplicates were removed using Picard tools, and data were imported into SeqMonk (www.bioinformatics.babraham.ac.uk/) and filtered for a minimum mapping quality of 20 or above. Reads were quantified at the gene level with the SeqMonk RNA-seq quantification pipeline, and raw counts were generated. Differential expression analysis was performed using DESeq2 (*105*). For the heatmap, scatterplot, and volcano plots, read counts were quantified at the gene level and log_2_-transformed with the SeqMonk RNA-seq quantification pipeline. Principal component analysis, heatmap, and scatterplots were generated using all genes with a count above 10 in at least four samples (out of 12 in total). The volcano plot was made using the same list, plotting the −log_10_-transformed false discovery rate (FDR) from the DESeq2 analysis against the log_2_ fold change of *Apc^+/−^;Bmal1^−/−^* relative to the other three genotypes. GSEA of Kyoto Encyclopedia of Genes and Genomes (KEGG) pathways was performed on the unfiltered differentially expressed gene list using the Bioconductor and clusterProfiler packages in R software (*106*). Pathways in GSEA were considered significant based on a *P* value cutoff of <0.05. Pathway analysis was visualized as a dot plot using the ggplot2 package in R. Gene ratio was calculated as the count of genes in the differentially expressed gene list that belong to a given set and divided by the setSize. SetSize is the total number of genes in the given KEGG gene set.

### Western blot

Organoid pellets were lysed in radioimmunoprecipitation assay lysis buffer [50 mM tris (pH 8), 150 mM NaCl, 5 mM EDTA, 15 mM MgCl_2_, and 1% NP-40] containing protease and phosphatase inhibitors [1× complete EDTA free cocktail tablet (Sigma-Aldrich), 0.5 mM phenylmethylsulfonyl fluoride, 20 mM NaF, 1 mM Na_3_VO_4_, 1 μM trichostatin A, and 10 mM nicotinamide]. Protein lysates were resolved on an SDS–polyacrylamide gel electrophoresis gel. Antibodies used for Western blot were the following: c-MYC (Abcam, ab32072), p84 (GeneTex, GTX70220), BMAL1 (Abcam, ab93806), and Apc clone FE9 (Sigma-Aldrich, MABC202).

### gDNA preparation from organoids

Organoid pellets were resuspended in Tris EDTA (TE) buffer supplemented with SDS and ribonuclease A and incubated at 37°C for 2 hours. Samples were then incubated at 55°C for 5 hours with 100 μg of proteinase K. PCI (25:24:1 ratio of phenol, chloroform, and isoamyl alcohol) was added, and the aqueous phase was combined with chloroform. Aqueous phase was transferred to a fresh 1.5-ml tube and then 1:10 volume of 3 M sodium acetate and 1 ml of ice-cold 100% ethanol were added. Samples were incubated overnight at −20°C and then centrifuged. DNA pellets were washed with ice-cold 70% ethanol and then air-dried. Pellets were resuspended in water.

### Digital polymerase chain reaction

gDNA was digested with high-fidelity Eco RI and Hind III overnight at 37°C. Sample master mix was prepared by combining 1× Absolute Q DNA dPCR Master Mix (Thermo Fisher Scientific), 900 nM forward and reverse primers, 250 nM FAM and HEX probe, and 1 ng of digested gDNA. *Apc* primers and FAM-labeled probe (Thermo Fisher Scientific) were used as the target assay. Reference primers and HEX probe targeting *ApoB* (Bio-Rad) were used as a reference for intestinal organoids from all four genotypes, and the *Tfrc* locus was used as a reference (Thermo Fisher Scientific, 4458366) for tumor and surrounding organoids. The master mix was loaded onto the Absolute Q microfluidic array partitioning plate, and the isolation buffer (Thermo Fisher Scientific) was overlaid. The dPCR plate was run in the Applied Biosystems QuantStudio Absolute Q dPCR System with a program of 96°C for 10 min, followed by 45 cycles at 96°C for 5 s and 60°C for 30 s. This dPCR system has been described in detail previously (*107*). Primer and probe information for dPCR are listed in table S1.

### WES analysis

Library preparation and 150-bp paired-end sequencing of gDNA, using selected primers to enrich for exomes, was performed by Novogene. Raw reads were aligned to the current mouse genome (GRCm39) using Burrows Wheeler Aligner Maximal Exact Match (BWA-MEM) (*108*), and PCR duplicates were removed using Picard tools. Variant calling was performed on matched early versus late organoids using Mutect2 and annotated using GATK functional annotator. Deduplicated, aligned reads were imported into SeqMonk, filtering out reads with a mapping quality of less than 20 and excluding read pairs further apart than 1000 bp. Tiled quantifications (200 bp) were generated, normalized to the largest data store, and shown in the browser view of the *Apc* locus. Reads were then quantitated over exons, normalized to the largest data store, corrected for probe length, and log-transformed. A scatterplot of the combined replicates of early- and late-passage organoids was generated. Statistical filtering was performed on normalized quantifications using Limma with a *P* value threshold of <0.01 (*109*). A volcano plot was generated using −log_10_-transformed FDR values from the Limma test against log_2_ fold change of late passage relative to early passage.

### Lentiviral transduction of organoids

The third-generation lentiviral packaging system was used for transduction of intestinal organoids. For production of viral particles, HEK293T cells were transfected with plasmids encoding lentiviral packaging (pRSV-Rev and pMDLg/pRRE), lentiviral envelope (pMD2.G), and the desired transfer vector. Lentivirus-containing medium was harvested 48 hours after transfection, filtered through a 0.45-μm syringe filter, and concentrated over 100-fold in volume by ultracentrifugation. For transduction of organoids, a previously described method was used, whereby organoids were overlaid with virus and then sandwiched in Matrigel (*110*). For the lentiviral transduction of *c-Myc* shRNA in *Apc^+/−^;Bmal1^−/−^* organoids, we used Lenti-sh1368 (Addgene, #29435).

### Fluorescence lifetime imaging microscopy

Fluorescence lifetime images of NADH were acquired with a Zeiss LSM 880 confocal microscope equipped with a 40× 1.2 numerical aperture C-Apochromat water-immersion objective attached to an A320 FastFLIM acquisition system (ISS). A titanium sapphire laser (Spectra-Physics Mai Tai) with a repetition rate of 80 MHz was used for two-photon excitation at a wavelength of 740 nm. The excitation and emission signals were separated by a 690-nm dichroic mirror. The NADH signal was collected by passing the emission signal through a bandpass filter of 460/80 nm and was detected via an external photomultiplier tube (H7522P-40, Hamamatsu). Organoids were imaged within a stage-top incubator kept at 5% CO_2_ and 37°C. FLIM data were acquired and analyzed with the software SimFCS 4, which was developed at the Laboratory for Fluorescence Dynamics at University of California, Irvine. To account for the instrument response function, FLIM images of coumarin 6 (~10 μM), which has a known lifetime of 2.5 ns in ethanol, were acquired. Organoids were manually segmented to only quantify FLIM pixels within the cellular regions of the organoids, avoiding background and luminal regions.

### Patient-derived colon isolation and organoid culture

Colonic human tissue was collected following surgical resection with informed consent and Institutional Review Board approval at St. Joseph Hospital Orange (Orange, CA). All patients were diagnosed with CRC, and the diagnosis was confirmed by a pathologist. Tumor tissue was separated from the surrounding normal colon by a pathologist, enabling the establishment of matched normal and tumor PDOs. Establishment and culture of PDOs were performed on the basis of previous publications (*111*–*113*). Briefly, tumor tissue was diced into small pieces and then dissociated in advanced DMEM/F12 [supplemented with P/S, 10 mM Hepes, 2 mM GlutaMAX, Primocin (100 μg/ml), collagenase II (1.5 mg/ml), hyaluronidase (20 μg/ml), and Y-27632] at 37°C for 1 hour, followed by vigorous shaking. For normal colon, muscle, fat, and submucosal layers were removed, and crypts were isolated with vigorous mechanical shaking after a 30-min dissociation rotating at 4°C [solution: PBS supplemented with 10 μM Y-27632, 4 mM EDTA, and DTT (200 μg/ml)]. For both normal and tumor organoids, cell isolates underwent filtration, red blood cell lysis treatment, and washes before being plated in Matrigel. Normal colon organoids were grown in advanced DMEM/F12 supplemented with 50% Wnt3a conditioned medium (from L-Wnt3a cells, provided by the laboratory of H. Clevers), 20% R-Spondin conditioned medium (from Cultrex Rspo1-expressing cells, Trevigen), 10% Noggin conditioned medium (from HEK293 cells stably transfected with pcDNA3 NEO mouse Noggin insert, provided by the laboratory of H. Clevers), 1× B27, 1.25 mM *N*-acetylcysteine, 10 mM nicotinamide, human EGF (50 ng/ml), 10 nM gastrin, 500 nM A83-01, 3 μM SB202190, 10 nM prostaglandin E2, 10 mM Hepes, 2 mM GlutaMAX, Primocin (100 μg/ml), and P/S. Tumor colon organoids were grown in the same medium but without the 50% Wnt3a conditioned medium. Medium was changed every 2 to 3 days, and organoids were passaged once per week following established protocols (*111*, *112*).

### Real-time luminescence recording of human colon PDOs

PDOs were transduced with pLV6-*Bmal1*-Luc (Addgene, #68833), which contains the nuclear receptor subfamily 1 group D member (NR1D1 or REV-ERB)/retinoic acid-related orphan receptor (ROR) response element regulatory motif. Forty-eight hours after transduction, PDOs were synchronized with dexamethasone (100 nM), supplemented with 0.5 mM beetle luciferin (Promega), and luminescence was measured. To analyze the data, the luminescence counts over time were analyzed using BioDare2 to determine the amplitude and period. First, circadian rhythmicity was examined using the JTK_CYCLE algorithm (*114*) implemented in BioDare2 according to the developer’s instructions (*115*). Briefly, luminescence counts over time from each PDO were detrended and tested by JTK_CYCLE using a cosine curve with 24-hour period as a preset. Rhythmicity was determined using a threshold *P* value cutoff of <0.001. All rhythmic data were then further analyzed to determine their amplitude and period using the fast Fourier transform nonlinear least squares function (*115*). If the estimated period was too short (less than 22 hours) or too long (more than 30 hours), the data were considered nonrhythmic. Last, BioDare2 was used to generate a heatmap, showing the rhythmicity of all samples tested.

### Metabolite extraction for metabolomics

Organoids were plated in glucose-free medium (Biowest) and supplemented with [U-^13^C]glucose (3151 mg/liter). Organoids were collected after 45 min in cold 40:20:20 MeOH:acetonitrile (ACN):H_2_O extraction solvent. Samples were immediately vortexed for 10 s and incubated at −20°C for 1 hour to facilitate protein precipitation. Samples were centrifuged at 16,000*g* for 15 min at 4°C, and supernatants were transferred to a fresh Eppendorf tube. Extracts were dried using a nitrogen evaporator (Organomation) before liquid chromatography–MS (LC-MS) processing. Protein concentrations were used to normalize LC-MS data.

### LC-MS metabolomics analysis

Dried metabolites were resuspended in 50% ACN:water, and 1/10 of the volume was loaded onto a Luna 3-μm NH2 100A (150 mm by 2.0 mm) column (Phenomenex). The chromatographic separation was performed on a Vanquish Flex (Thermo Fisher Scientific) with mobile phases A [5 mM NH_4_AcO (pH 9.9)] and B (ACN) at a flow rate of 200 μl/min. A linear gradient from 15% A to 95% A over 18 min was followed by 9 min of isocratic flow at 95% A and reequilibration to 15% A. Metabolites were detected with a Thermo Fisher Scientific Q Exactive mass spectrometer run with polarity switching (+3.5 kV/−3.5 kV) in full scan mode with an *m*/*z* (mass/charge ratio) range of 65 to 975. TraceFinder 4.1 was used to quantify the targeted metabolites by area under the curve using expected retention time and accurate mass measurements (<5 parts per million). Relative amounts of metabolites were calculated by summing up the values for all isotopologs of a given metabolite. Metabolite isotopolog distributions were corrected for natural ^13^C abundance (*116*).

### TCGA analysis

TCGA colon cancer sequencing data were downloaded from the UCSC Xena portal (*117*) using the genomic data commons (GDC) TCGA Colon Cancer colon adenocarcinoma (COAD) cohort and analyzed in R. Normalized sequencing reads were then filtered for genes present above 0 in >50% of individuals. Tumor and surrounding tissue (identified from the TCGA ID sample code) were then binned separately and analyzed. Comparison of normalized counts of clock genes was plotted using ggplot2 and compared for each circadian gene using two-way Student’s *t* test. Correlations between clock genes in tumor or surrounding tissue were assessed using midweight bicorrelations and associated regression *P* values using the package weighted gene co-expression network analysis (WGCNA) (*118*). Wnt signaling pathway genes were isolated from Gene Ontology (GO:0016055) and then correlated with clock genes using WGCNA. To assign principal component risk scores based on gene expression, all correlated Wnt clock genes were used as previously described (*85*–*87*). Specifically, principal components analysis was performed on tumor samples from all significant circadian-Wnt gene correlations using the R package FactoExtra, and all individuals were binned into 10 quantiles on the basis of principal component contributions. These contributions were then split in half, ranked by the absolute value of principal component contribution, where higher-contribution individuals were assigned “high covariance” and lower-contribution/variance individuals were assigned “low covariance.” Significance of survival differences between groups was calculated using a log-rank test. Survival curves were generated and analyzed using R packages survival and survminer. Relationships using Student’s *t* test or correlations were considered significant at *P* < 0.001.

## Notes

View/request a protocol for this paper from *Bio-protocol*.
