# Supplementary material for: Disruption of the circadian clock drives Apc loss of heterozygosity to accelerate colorectal cancer
Source: Sci Adv. 2022 Aug 10;8(31):eabo2389. doi: 10.1126/sciadv.abo2389 (PMC9365282; doi:10.1126/sciadv.abo2389)
Supplement: Supplementary file 1 — Figs. S1 to S10 Table S1 [file sciadv.abo2389_sm.pdf]

Science Advances
AAAS

Supplementary Materials for

Disruption of the circadian clock drives *Apc* loss of heterozygosity to accelerate colorectal cancer

Sung Kook Chun *et al.*

Corresponding author: Selma Masri, smasri@uci.edu

Sci. Adv. **8**, eabo2389 (2022)
DOI: 10.1126/sciadv.abo2389

This PDF file includes:

Figs. S1 to S10
Table S1

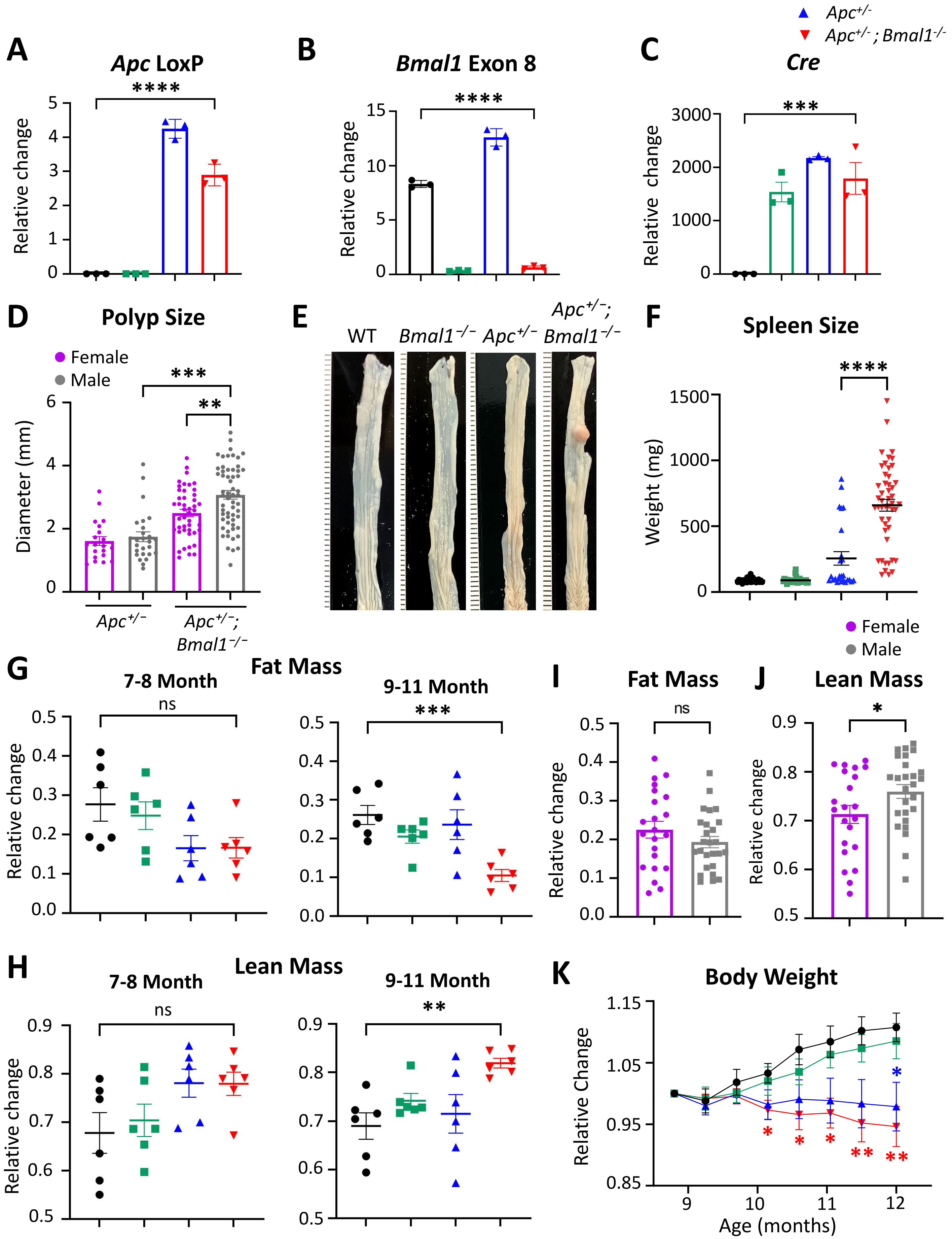
Supplementary Figure 1: Chun, Fortin, Fellows et al.
WT
Bmal1-/-
Apc+/-
Apc+/- ; Bmal1-/-
A
Apc LoxP
Relative change
B
Bmal1 Exon 8
C
Cre
D
Polyp Size
Female
Male
Diameter (mm)
E
WT
Bmal1-/-
Apc+/-
Apc+/-; Bmal1-/-
F
Spleen Size
Weight (mg)
G
Fat Mass
7-8 Month
9-11 Month
H
Lean Mass
I
Fat Mass
J
Lean Mass
K
Body Weight
Relative Change
Age (months)

Supplementary Figure 2: Chun, Fortin, Fellows et al.

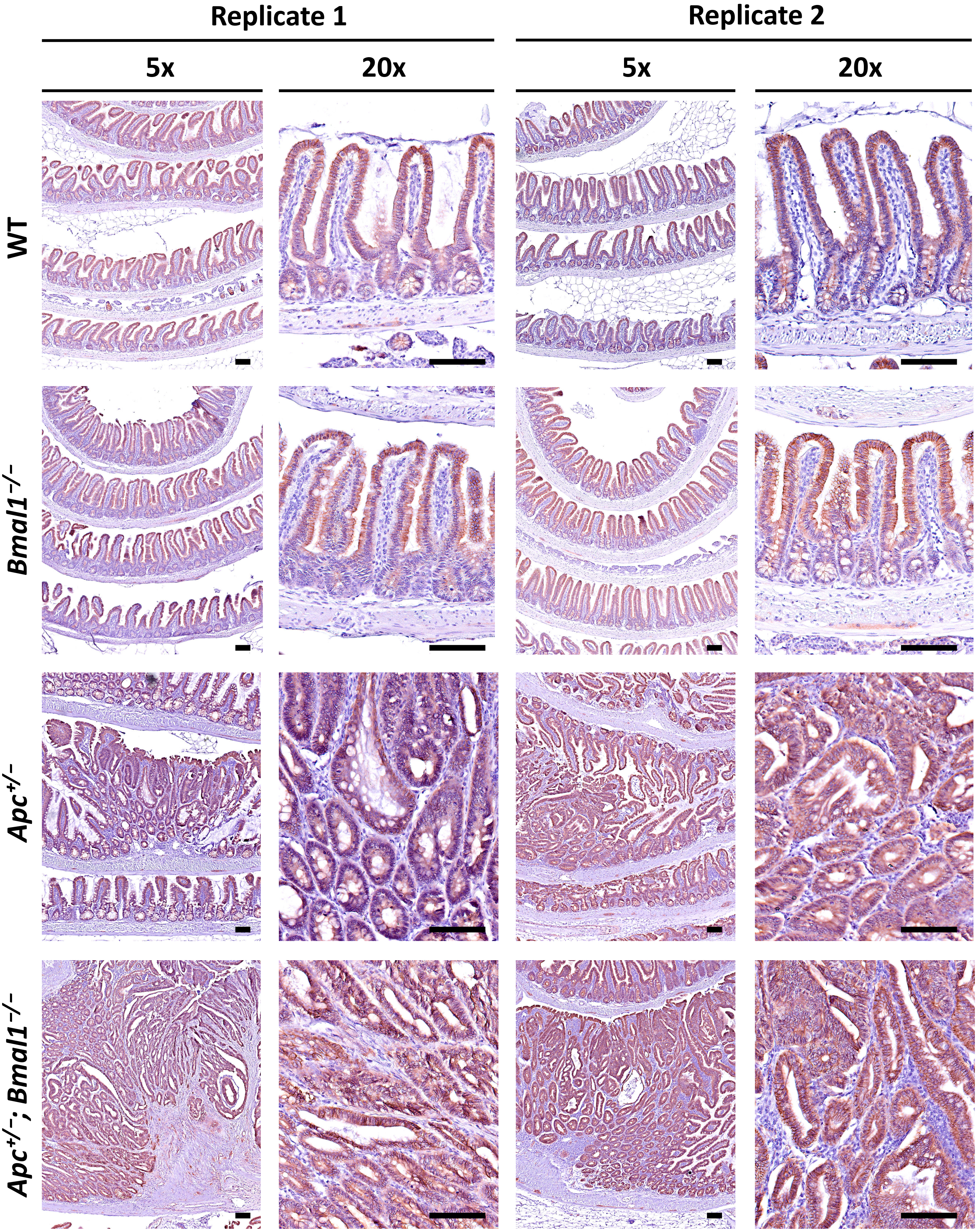

Supplementary Figure 3: Chun, Fortin, Fellows et al.

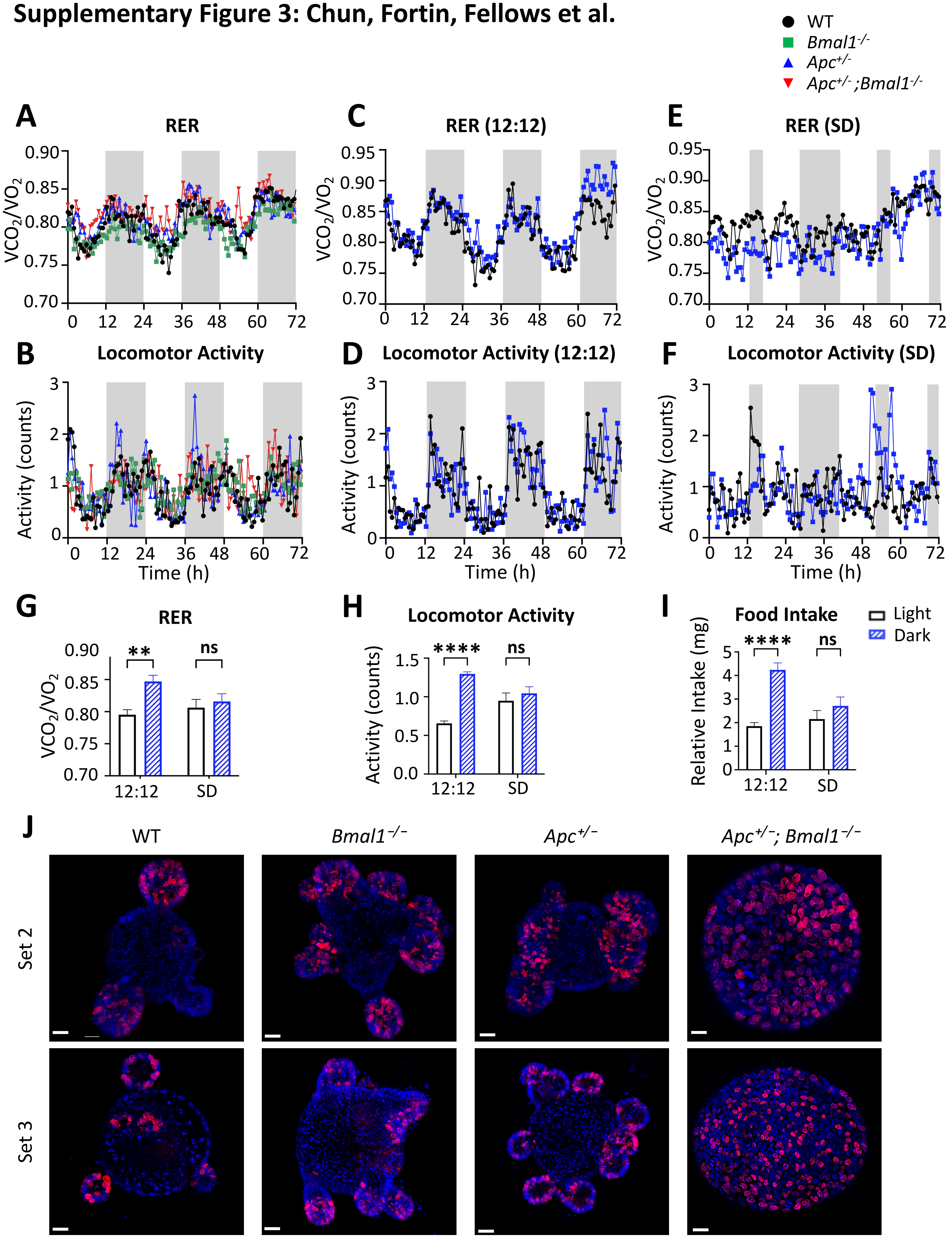

Supplementary Figure 4: Chun, Fortin, Fellows et al.

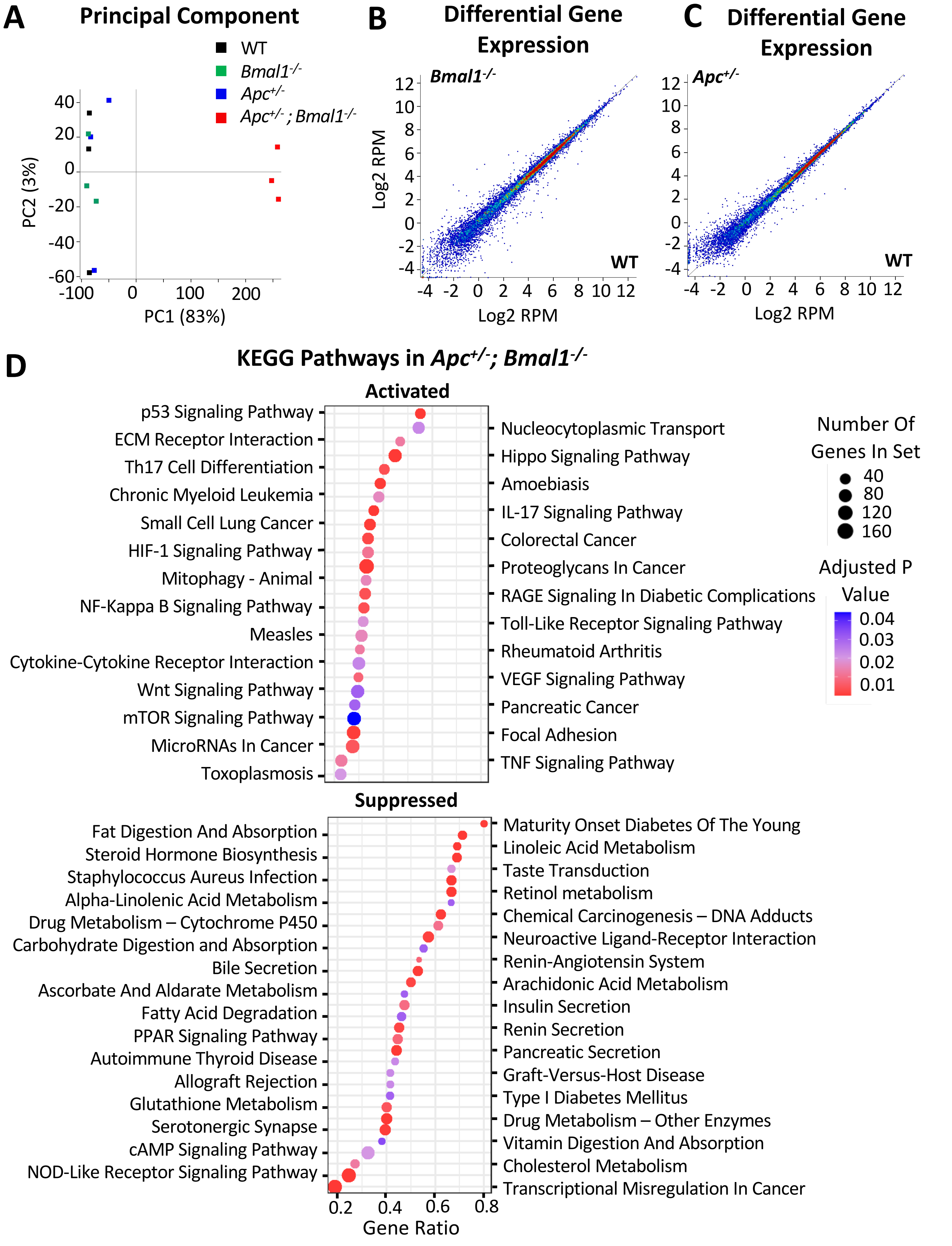

Supplementary Figure 5: Chun, Fortin, Fellows et al.

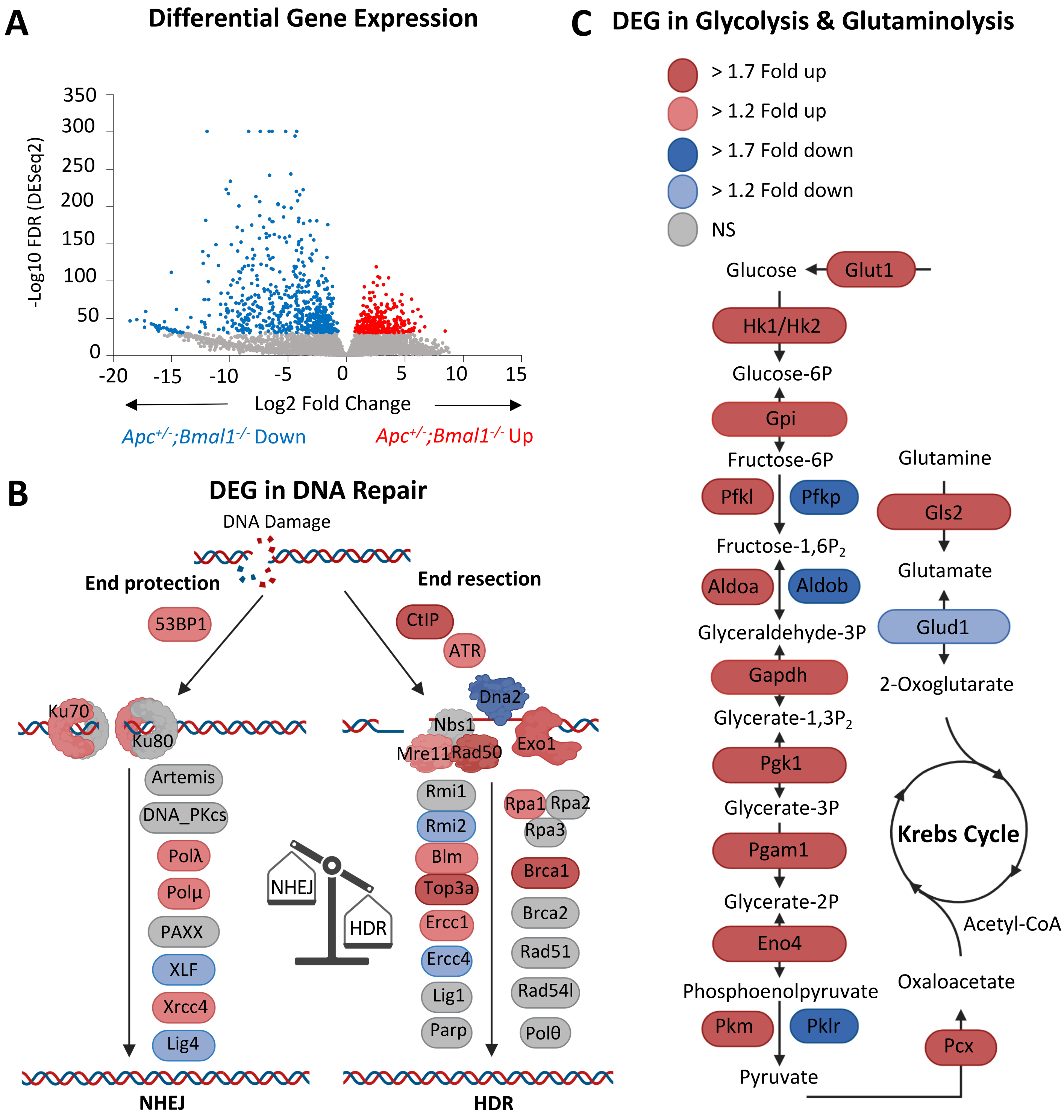

Supplementary Figure 6: Chun, Fortin, Fellows et al.

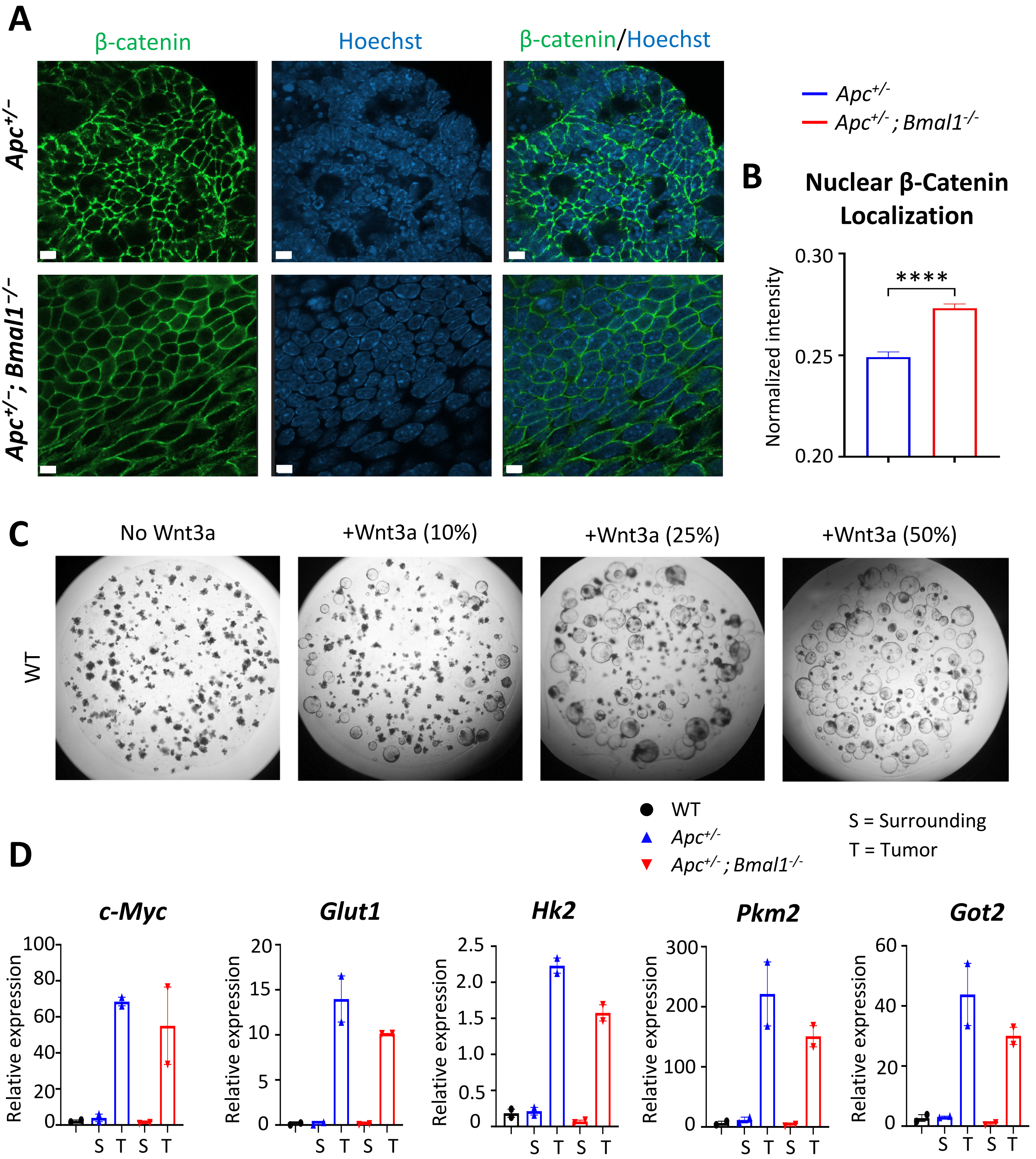

Supplementary Figure 7: Chun, Fortin, Fellows et al.

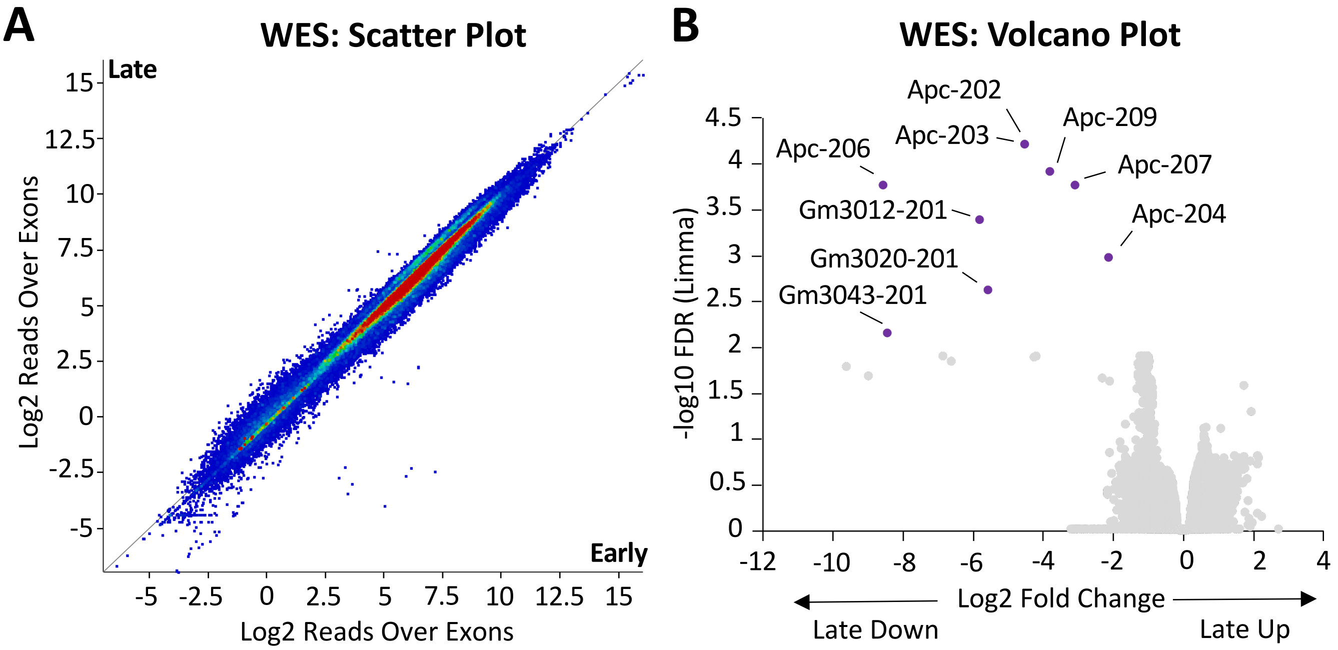

C

Gene	Mutations in Apc$^{+/-}$;Bmal1$^{-/-}$ Organoids				DEG	Signaling Pathway
	1	2	3	4		
Apc	-	-	-	-	Down	
Ctnnb1	-	-	-	-	-	Wnt
Axin2	-	-	-	-	Up	
Trp53	-	-	-	L302R	Up	Ret, G1/S checkpoint, apoptosis
Kras	-	-	-	-	Down	
Nras	-	-	-	-	Up	
Hras	-	-	-	-	Up	Ras/Raf/Mapk
Braf	-	A731V	-	-	-	
Raf1	-	-	-	-	Down	
Myc	-	-	-	V239G, T247P, S250R	Up	Notch, Erk and Mapk
Smad4	-	-	-	-	Up	TGF-β, Bmp
Tgfbr1	-	-	-	E106*	-	PI3K/AKT, TGF-β
Tgfbr2	-	-	-	-	Up	
Pik3ca	-	-	-	-	-	PI3K/AKT
Pten	-	-	-	S398T	Up	
Dcc	-	-	-	-	-	Netrin-1
Egfr	-	-	-	-	Up	RAS/RAF/MAPK and PI3K/AKT
Cdkn2a	-	-	-	-	Up	Cell cycle
Cdkn2b	-	-	-	-	Down	

Supplementary Figure 8: Chun, Fortin, Fellows et al.

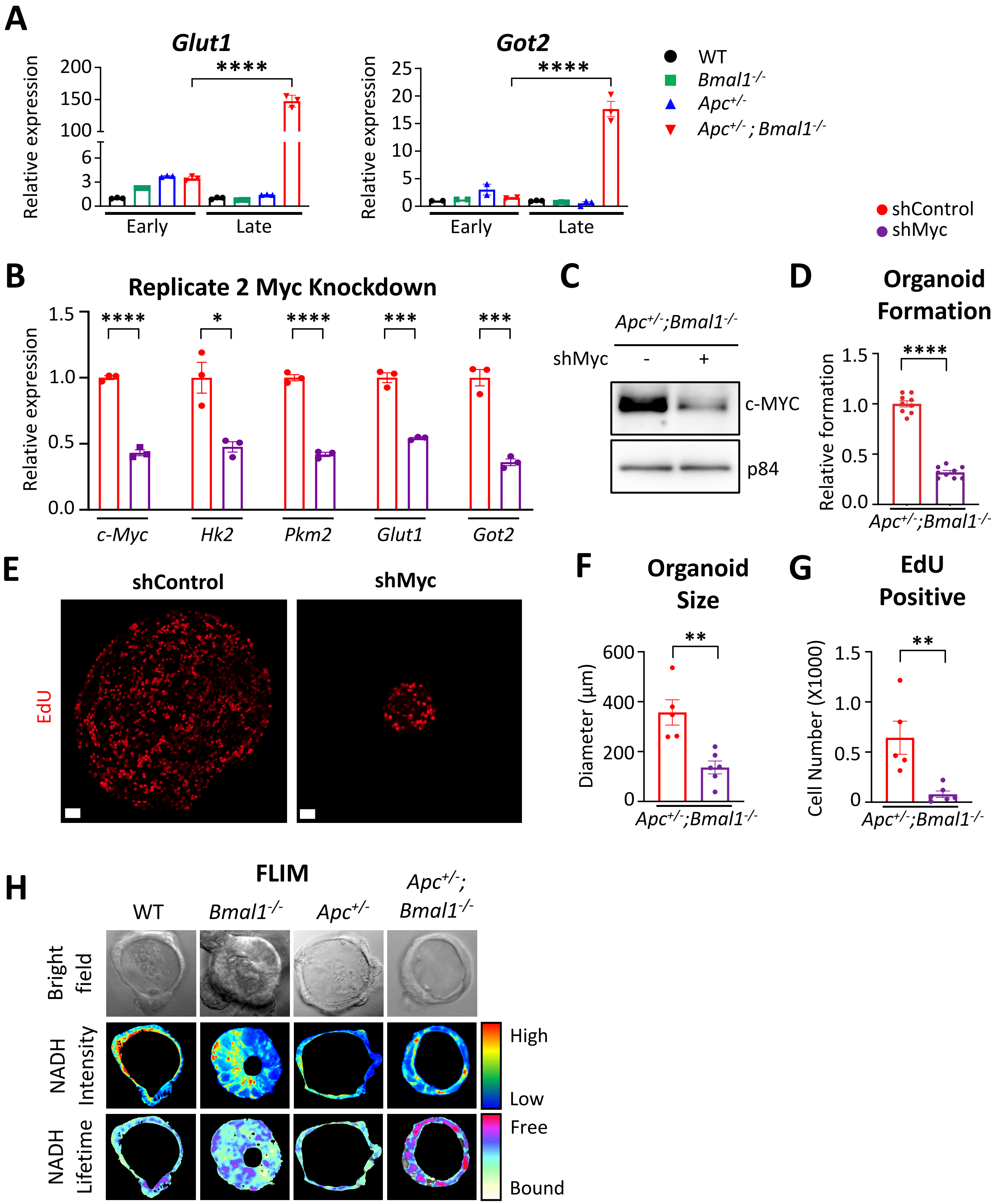

Supplementary Figure 9: Chun, Fortin, Fellows et al.

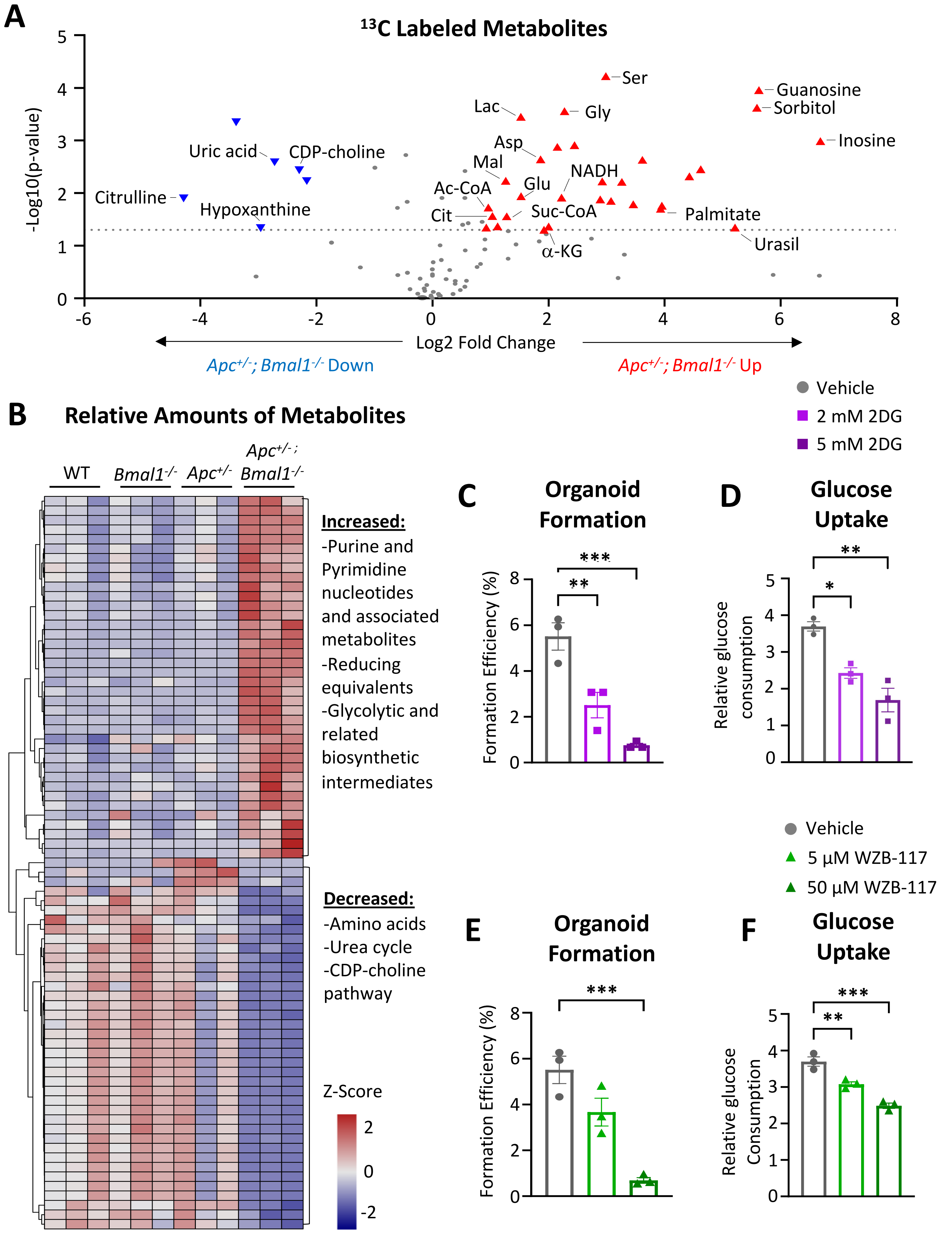

Supplementary Figure 10: Chun, Fortin, Fellows et al.

A

PDO #	Tumor type	Mutational profile		Age/ gender	Period		Amplitude (Tumor/Normal)
		MMR	Description		Normal	Tumor	
1	Adenocarcinoma	No loss	--	--	25.32	26.26	0.23
2	Adenocarcinoma	No loss	--	48/F	25.85	NR	0.17
3	Adenocarcinoma	No loss	--	69/F	26.18	NR	0.032
4	Adenocarcinoma	Loss	• MLH1 and PMS2: loss<br>• BRAF mutation is detected	--	26.38	NR	0.045
5	Adenocarcinoma with liver metastasis	No loss	• KRAS mutation is detected (c.38G>A(p.G13D)	83/M	26.88	NR	0.27
6	Adenocarcinoma	Loss	• MSH2: loss<br>• MSI: high<br>• BAT-25, BAT-26, MONO-27, NR-21, NR-24: unstable	35	27.89	NR	0.13
7	Adenocarcinoma	Loss	• MSH6: loss	--	25.09	27.19	0.20

B

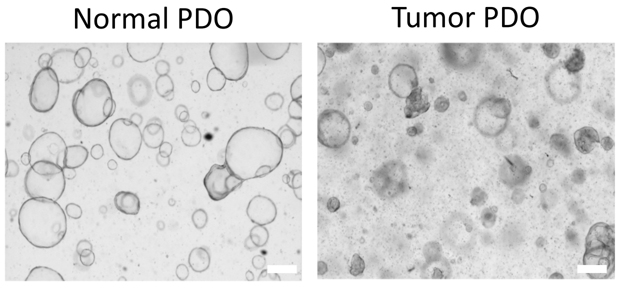

C

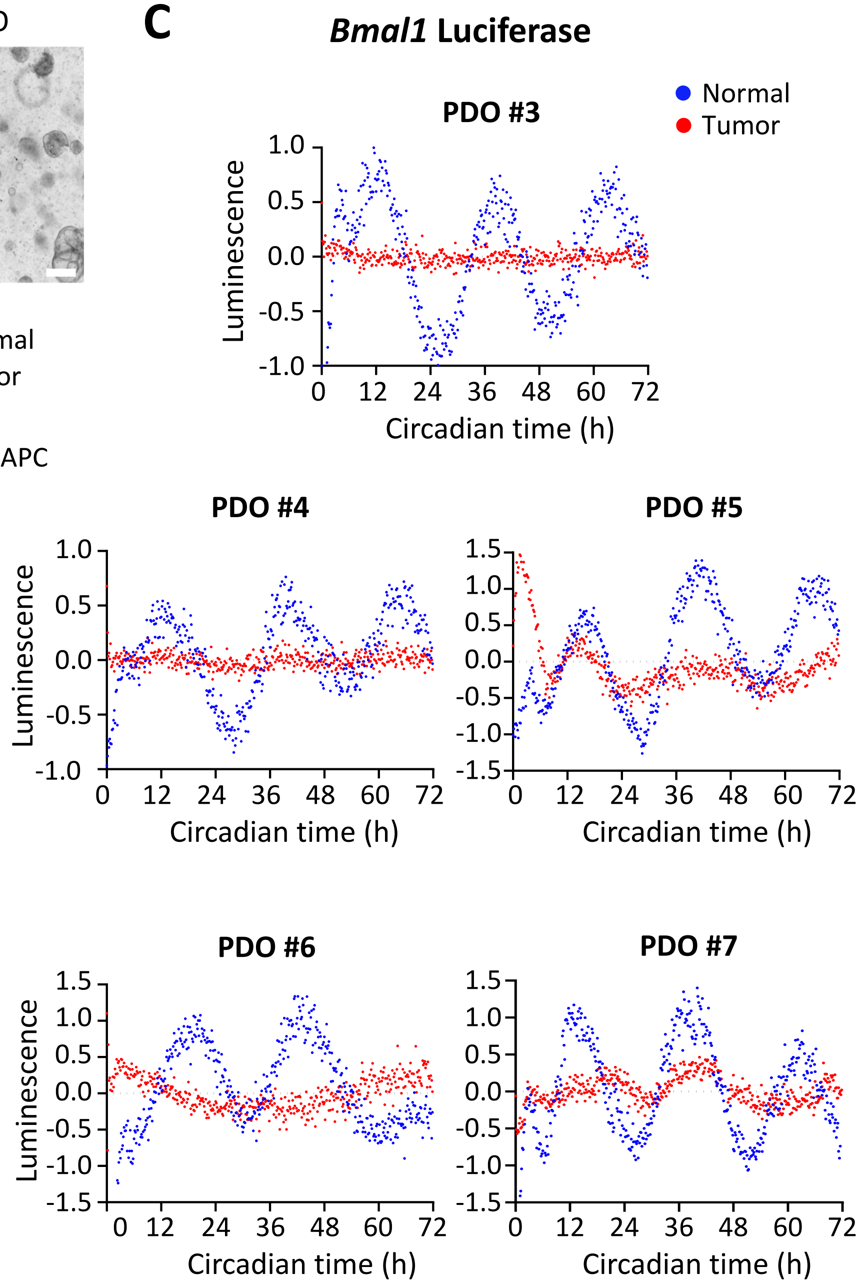

D

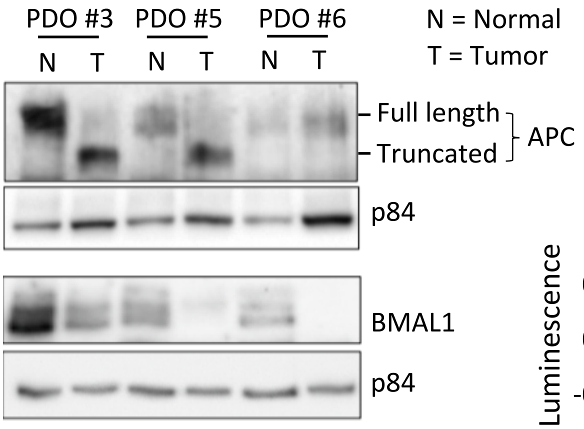

E

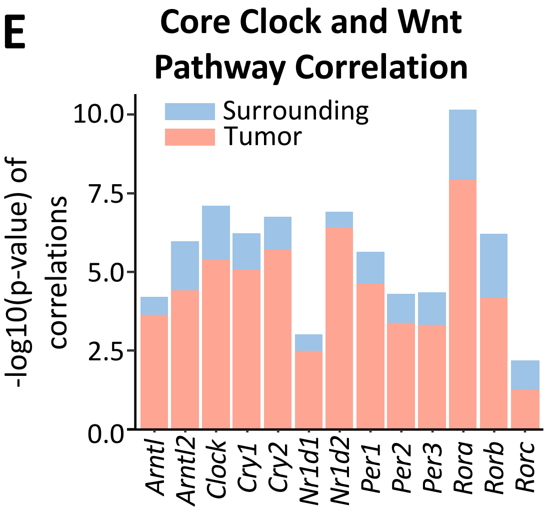

Table 1: Primer and Probe Information.

Primer and probe information used for qPCR and dPCR experiments.

**qPCR Primer Name**	**Primer Sequence**
Apc mRNA Fwd	5'-GCTCCTCGAAGGTTGACAAG-3'
Apc mRNA Rev	5'-CTGTCTTGGGAGCTGGACAT-3'
Axin2 mRNA Fwd	5'-TGTCCTGGGGGAACAGATTA-3'
Axin2 mRNA Rev	5'-TTTTGGCAAGGTACCACCTC-3'
C-myc mRNA Fwd	5'-AGCTCGCCCAAATCCTGTAC-3'
C-myc mRNA Rev	5'-TTGTGCTGGTGAGTGGAGAC-3'
Gls2 mRNA Fwd	5'-CAGGAGCGTATCCCTATCCA-3'
Gls2 mRNA Rev	5'-GCTGCATCTTGCTCATGC-3'
Got2 mRNA Fwd	5'-CGTTACCGAAGCCTTCAAGA-3'
Got2 mRNA Rev	5'-GGGCAGGTATTCTTTGTCCA-3'
Hk2 mRNA Fwd	5'-CAACTCCGGATGGGACAG-3'
Hk2 mRNA Rev	5'-CACACGGAAGTTGGTTCCTC-3'
Pkm2 mRNA Fwd	5'-CTCCTTCAAGTGCTGCAGTG-3'
Pkm2 mRNA Rev	5'-CAGTCTGGGGATTTCGAGTC-3'
Slc2a1 mRNA Fwd	5'-TTGTTGTAGAGCGAGCTGGA-3'
Slc2a1 mRNA Rev	5'-ACCAGGGCCTACTTCAAAGA-3'
Slc7a5 mRNA Fwd	5'-ATGACGCTGATGTACGCCTT-3'
Slc7a5 mRNA Rev	5'-CGGAGCCACATCATACCGAT-3'
Survivin mRNA Fwd	5'-GAACCCGATGACAACCCGAT-3'
Survivin mRNA Rev	5'-TTGGCTCTCTGTCTGTCCAG-3'
Wnt3a mRNA Fwd	5'-ACACTTGAGCAGAACGGATACA-3'
Wnt3a mRNA Rev	5'-TGGATACAGCAGGTTGGTAGG-3'
Apc Lox P Fwd	5'-ATGTACCTGACTGATGAAGTTCCTA-3'
Apc Lox P Rev	5'-CCTCGAGGTCGACGGTAT-3'
Bmal1 Exon 8 Fwd	5'-AGGCCCACAGTCAGATTGAA-3'
Bmal1 Exon 8 Rev	5'-GCTGAACAGCCATCCTTAGC-3'
Cre Fwd	5'-CCATCTGCCACCAGCCAG-3'
Cre Rev	5'-TCGCCATCTTCCAGCAGG-3'
Per1 Fwd	5'-ATCCTGATCGCATTGGCTGACTGA-3'
Per1 Rev	5'-TCTCTTCCTGGCATCTGATTGGCT-3'

**dPCR Primer/Probe Name**	**Primer Sequence**
Apc Fwd	5’-GAAAGTGGAGGTGGGATA-3’
Apc Rev	5’ GTGGTCTTCGTTTGTAGC-3’
Apc FAM Probe	5’-/56-FAM/GGAA+TGTGTCCAGCT+TGA/3IABkFQ/-3’
ApoB Primer Probe Mix (Biorad)	Region: Mm39|chr18:34354047-34354169
Tfrc Primer Probe Mix (Thermo Fisher Scientific)	Region: Mm37|chr16:32626732-32626823

Supplementary Figure legends

Supplementary Figure 1: Disruption of *Bmal1* accelerates intestinal tumorigenesis *in vivo*. (A) Verification of loxP sites within the *Apc* locus by qPCR using gDNA isolated from IECs of WT, $Bmal1^{-/-}$, $Apc^{+/-}$, and $Apc^{+/-}$;$Bmal1^{-/-}$ mice. **(B)** qPCR confirmation of *Bmal1* exon 8 floxed allele using IEC gDNA. **(C)** Validation of Cre using qPCR in IEC gDNA. **(D)** Scatter plot of small intestinal polyp sizes from $Apc^{+/-}$ and $Apc^{+/-}$;$Bmal1^{-/-}$ mice divided by gender (n=4 mice/gender/genotype). **(E)** Representative images of linearized colon tissue from all GEMMs. **(F)** Scatter plot of spleen weights from 28 WT, 31 $Bmal1^{-/-}$, 25 $Apc^{+/-}$, and 48 $Apc^{+/-}$;$Bmal1^{-/-}$ mice. **(G)** Fat mass and **(H)** lean mass measurements relative to body weight from 7-8 month or 9-11 month old mice (n=6 mice/genotype). **(I)** Fat mass and **(J)** lean mass relative to body weight for each gender (n=22 females, n= 26 males). **(K)** Body weight measurements of mice normalized to individual mouse starting weight (n=10 mice/genotype). Statistical significance was determined by one-way ANOVA for G and H, Student's unpaired t-test for I and J, and two-way ANOVA with Tukey's multiple comparisons for K. For K, significant multiple comparisons for WT versus $Apc^{+/-}$;$Bmal1^{-/-}$ are shown in red and WT versus $Apc^{+/-}$ are shown in blue. Asterisks represent p-values from the t-test or multiple comparisons, with * indicating a p-value of < 0.05, ** a p-value of < 0.01, *** a p-value of < 0.001, **** a p-value of < 0.0001, and ns = not significant.

Supplementary Figure 2: β-Catenin IHC staining *in vivo.* IHC was performed for β-Catenin with a hematoxylin counterstain on sectioned small intestinal swiss rolls from WT, $Bmal1^{-/-}$, $Apc^{+/-}$, and $Apc^{+/-}$;$Bmal1^{-/-}$ mice. 5x and 20x magnifications are shown for two biological replicates for each of the four genotypes. Scale bar = 100 µm.

Supplementary Figure 3: Indirect calorimetry of GEMM and cellular analysis of organoid model. (A) RER and **(B)** locomotor activity generated by Phenomaster metabolic cages from mice. WT n=4, $Bmal1^{-/-}$ n=6, $Apc^{+/-}$ n=5, and $Apc^{+/-}$;$Bmal1^{-/-}$ n=4. **(C)** RER and **(D)** locomotor activity from WT and $Apc^{+/-}$ mice maintained in 12:12 conditions. WT n=6, $Apc^{+/-}$ n=6. **(E)** RER and **(F)** locomotor activity from WT and $Apc^{+/-}$ mice maintained in SD conditions. WT n=6, $Apc^{+/-}$ n=6. Quantification

of **(G)** RER, **(H)** locomotor activity, and **(I)** food intake normalized to body weight of 12:12 and SD mice. **(J)** EdU incorporation in WT, *Bmal1*$^{-/-}$, *Apc*$^{+/-}$, and *Apc*$^{+/-}$;*Bmal1*$^{-/-}$ organoids (n=2 independent organoid lines). Merged channels of EdU (red) and Hoechst (blue) were taken on Zeiss Elyra 7 Super-Resolution Confocal Microscope. Scale bar = 20 µm. Data represent the mean ± SEM and statistical significance was determined by one-way ANOVA with Tukey's multiple comparisons. Asterisks represent p-values from the multiple comparisons, with ** indicating a p-value of < 0.01, **** a p-value of < 0.0001, and ns = not significant.

Supplementary Figure 4: Transcriptome analysis of organoid system. RNA-Seq was performed using late-stage WT, *Bmal1*$^{-/-}$, *Apc*$^{+/-}$, and *Apc*$^{+/-}$;*Bmal1*$^{-/-}$ organoids (n=3 independent organoid lines per genotype). **(A)** Principal component analysis showing the variation in overall gene expression between replicate organoid lines from all four genotypes. Scatter plot showing expression of transcripts in **(B)** *Bmal1*$^{-/-}$ or **(C)** *Apc*$^{+/-}$ compared to WT. Quantified reads are normalized per million mapped reads and log2 transformed (log2 RPM). **(D)** Significantly activated and suppressed KEGG pathways in *Apc*$^{+/-}$;*Bmal1*$^{-/-}$ relative to the other three genotypes as determined by GSEA. Analysis is visualized as a dot plot with point size reflecting the number of genes in a KEGG pathway term and gene ratio as the count of differentially expressed genes in a given KEGG pathway term.

Supplementary Figure 5: Pathway analysis of organoid transcriptome. (A) Volcano plot showing -log10 FDR against log2 fold change of *Apc*$^{+/-}$;*Bmal1*$^{-/-}$ relative to the other three genotypes. Genes that are suppressed in *Apc*$^{+/-}$;*Bmal1*$^{-/-}$ with a -log10 FDR greater than 30 are highlighted in red, the genes that are activated in *Apc*$^{+/-}$;*Bmal1*$^{-/-}$ with the same FDR threshold are highlighted in blue. **(B)** Differential gene expression in NHEJ and HDR pathways. **(C)** Differential gene expression in glycolysis and glutaminolysis pathways. Genes are colored according to the DESeq2 analysis of the three genotypes versus *Apc*$^{+/-}$;*Bmal1*$^{-/-}$. The threshold for significance was an FDR less than 0.05.

Supplementary Figure 6: Analysis of Wnt/β-Catenin signaling in organoids. (A) β-Catenin IF of *Apc*$^{+/-}$ and *Apc*$^{+/-}$;*Bmal1*$^{-/-}$ organoids. Scale bar = 5 μM. **(B)** Quantification of nuclear β-Catenin from organoid IF (n=10,470 cells for *Apc*$^{+/-}$ and 12,822 cells for *Apc*$^{+/-}$;*Bmal1*$^{-/-}$). **(C)** Microscope images of WT organoids grown in ENR media supplemented with 10, 25, or 50% Wnt3a-conditioned media for 5 days. **(D)** Gene expression analysis of *c-Myc* and the *c-Myc* targets *Glut1, Hk2*, *Pkm2*, and *Got2* in organoids derived from tumor and normal surrounding epithelium (n=2 independent organoid lines). Data represent the mean ± SEM. Significance was determined using Student's unpaired t-test. Asterisks represent p-values with **** indicating a p-value of < 0.0001.

Supplementary Figure 7: Clock disruption accelerates *Apc* loss of heterozygosity. (A) WES from early and late passage *Apc*$^{+/-}$;*Bmal1*$^{-/-}$ organoids. Scatter plot shows log2 read counts over exons of early versus late passage (n=4 independent organoid lines). **(B)** Volcano plot showing -log10 FDR against log2 fold change of late relative to early passage *Apc*$^{+/-}$;*Bmal1*$^{-/-}$ organoids. Transcripts that change in exon read depth in late with a -log10 FDR of 2 or more are highlighted in purple. **(C)** Point mutations in genes frequently mutated in colorectal cancer as identified using variant calling of matched early versus late passage *Apc*$^{+/-}$;*Bmal1*$^{-/-}$ organoids. A dash indicates where no mutations were identified in that gene for that organoid line. Significant DEGs as identified by RNA-seq with an FDR threshold of 0.05 are indicated.

Supplementary Figure 8*: c-Myc* activation in *Apc*$^{+/-}$;*Bmal1*$^{-/-}$ organoids (A) Gene expression analysis of *Glut1* and *Got2* in early and late-stage WT, *Bmal1*$^{-/-}$, *Apc*$^{+/-}$, and *Apc*$^{+/-}$;*Bmal1*$^{-/-}$ organoids. shRNA mediated knockdown of *c-Myc* in *Apc*$^{+/-}$;*Bmal1*$^{-/-}$ organoids. **(B)** Gene expression analysis of *c-Myc*, *Hk2*, *Pkm2*, *Glut1*, and *Got2* by qPCR (n = 3 independent experiments). **(C)** Knockdown of *c-Myc* protein abundance validated by western blot and shown relative to p84 protein abundance. **(D)** Effect of *c-Myc* knockdown on organoid formation in *Apc*$^{+/-}$;*Bmal1*$^{-/-}$ organoids. Formation is presented as fold change relative to the shRNA control infection (n = 9 independent experiments). **(E)** Representative images of EdU staining in control and shMyc organoids. **(F)** Quantification of size in control and shMyc organoids. **(G)** Quantification of EdU positive cells in control and shMyc organoids. **(H)** Representative images of target organoids

showing bright field (upper panel), NADH intensity (middle panel) and NADH lifetime (lower panel) signal measured by fluorescence-lifetime imaging microscopy (FLIM) in WT, *Bmal1*$^{-/-}$, *Apc*$^{+/-}$, and *Apc*$^{+/-}$;*Bmal1*$^{-/-}$ organoids. Data represent the mean ± SEM and statistical significance was determined using one-way ANOVA with Tukey's multiple comparisons for A and Student's unpaired t-test for B, D, F, and G. Asterisks represent p-values from the t-test or multiple comparisons, with * indicating a p-value of < 0.05, ** a p-value of < 0.01, *** a p-value of < 0.001, and **** a p-value of < 0.0001.

Supplementary Figure 9: Disruption of *Bmal1* rewires cellular metabolism in organoids. (A) Volcano plot showing -log10 p-value against log2 fold change of ^{13}C labeled metabolites in *Apc*$^{+/-}$;*Bmal1*$^{-/-}$ relative to WT. Significantly decreased metabolites are highlighted blue, while significantly increased metabolites are in red. **(B)** Heatmap of significantly altered metabolites from untargeted metabolomics analysis in WT, *Bmal1*$^{-/-}$, *Apc*$^{+/-}$, and *Apc*$^{+/-}$;*Bmal1*$^{-/-}$ organoids (n=3 independent organoid lines/genotype). Each column represents an individual organoid line, and each row represents a unique metabolite. Enrichment of each metabolite in a single sample is depicted according to the color scale. Hierarchical clustering is indicated on the left side and key groups of metabolites are indicated on the right. **(C)** Organoid formation and **(D)** glucose uptake assays of organoids treated with 2DG. **(E)** Organoid formation and **(F)** glucose uptake assays of organoids treated with WZB-117. Data represent the mean ± SEM and significance was determined using one-way ANOVA with Tukey's multiple comparisons. Asterisks represent p-values from the multiple comparisons, with * indicating a p-value of < 0.05, ** a p-value of < 0.01 and *** a p-value of < 0.001.

Supplementary Figure 10: Analysis of circadian rhythms in human colon tissue. (A) Pathological information, mutational profile and, if available, age and gender from seven patients whose colon tissue was used to create PDOs. *Bmal1*-driven luminescence monitoring of normal and tumor PDOs was used to determine period along with tumor amplitude relative to normal PDO. Amplitudes and periods were calculated using BioDare2. NR = non-rhythmic **(B)** Bright field microscopy images of organoids established from normal and tumor tissue from the same

patient. Scale bar = 100 µm. **(C)** Detrended *Bmal1*-driven real-time bioluminescence over 72 hours from matched normal and tumor PDOs. **(D)** APC and BMAL1 protein abundance in tumor and normal matched PDOs relative to p84 abundance. **(E)** The average -log10(p-value) for regressions between core clock and Wnt pathway genes. P-values were estimated based regressions from the on midweight bicorrelation coefficient.
